# Diversity of flavour characteristics of table grapes and their contributing volatile compounds analysed by the solvent-assisted flavour evaporation method

**DOI:** 10.1093/hr/uhae048

**Published:** 2024-02-26

**Authors:** Kazuki Moriyama, Atsushi Kono, Ryusuke Matsuzaki, Akifumi Azuma, Noriyuki Onoue, Yoshihiko Sekozawa, Akihiko Sato, Sumiko Sugaya

**Affiliations:** Graduate School of Life and Environmental Sciences, University of Tsukuba, 1-1-1 Tennodai, Tsukuba, Ibaraki 305-8572, Japan; Institute of Fruit Tree and Tea Science, National Agriculture and Food Research Organization (NARO), 301-2 Mitsu, Akitsu, Higashihiroshima, Hiroshima 739-2494, Japan; Institute of Fruit Tree and Tea Science, NARO, 2-1 Fujimoto, Tsukuba, Ibaraki 305-8605, Japan; Institute of Fruit Tree and Tea Science, National Agriculture and Food Research Organization (NARO), 301-2 Mitsu, Akitsu, Higashihiroshima, Hiroshima 739-2494, Japan; Institute of Fruit Tree and Tea Science, National Agriculture and Food Research Organization (NARO), 301-2 Mitsu, Akitsu, Higashihiroshima, Hiroshima 739-2494, Japan; Department of Intellectual Property, NARO, 3-1-1 Kannondai, Tsukuba, Ibaraki 305-8517, Japan; Institute of Fruit Tree and Tea Science, National Agriculture and Food Research Organization (NARO), 301-2 Mitsu, Akitsu, Higashihiroshima, Hiroshima 739-2494, Japan; Institute of Life and Environmental Sciences, University of Tsukuba, 1-1-1 Tennodai, Tsukuba, Ibaraki 305-8572, Japan; Institute of Fruit Tree and Tea Science, National Agriculture and Food Research Organization (NARO), 301-2 Mitsu, Akitsu, Higashihiroshima, Hiroshima 739-2494, Japan; Experimental Farm, Kindai University, 2355-2 Yuasa, Yuasa, Wakayama 643-0004, Japan; Institute of Life and Environmental Sciences, University of Tsukuba, 1-1-1 Tennodai, Tsukuba, Ibaraki 305-8572, Japan

## Abstract

To identify the compounds that contribute to the diverse flavours of table grapes, the flavours and volatile compounds of 38 grape cultivars harvested over 3 years are evaluated through sensory analysis and solvent-assisted flavour evaporation (SAFE). The cultivars are characterized and grouped into seven clusters by hierarchical cluster analysis (HCA) using sensory evaluation data with a flavour wheel specific to table grapes. These clusters were similar to conventional flavour classifications, except that the foxy and neutral cultivars form multiple clusters, highlighting the flavour diversity of table grapes. The SAFE method provides a comprehensive profile of the volatile compounds, including slightly volatile compounds whose profiles are lacking in hybrid grapes and *Vitis rotundifolia*. The sensory evaluation is supported by the volatile compound profiles, and relationships between the datasets are clarified by multivariate analysis. Specific accumulations and combinations of compounds (α-pinene, β-pinene, phenylethyl alcohol, furaneol, mesifurane, methyl *N*-formylanthranilate, and mixed ethyl ester and monoterpenoid) were also identified that contribute to the diversity of flavours (fresh green, floral, fruity, fatty green, sweet, fermented/sour) in table grapes, including linalool and linalool analogues (muscat flavour) along with ethyl ester and hydroxyethyl esters (foxy flavour). The accumulation of these compounds was positively related to a higher flavour intensity. Their specific accumulation and combination supported the flavour diversity of table grapes. This study identified novel flavour-associated compound profiles in table grapes through in-depth volatile compound analysis and non-conventional multivariate analysis.

## Introduction

*Vitis vinifera* are the most commonly cultivated grapes, with a global cultivation area of 7.5 million ha, half of which is used to produce wine [1]. However, interspecific hybrids (especially the hybrids obtained from *Vitis labrusca* L. and *V. vinifera* L.) and the related genus Muscadinia (*Vitis rotundifolia*) are grown for wine and table grapes in regions with an unfavourable climate or disease pressure [2].

The taste and aroma of various fruits give rise to distinct flavours [3]. Flavour is an integral property of both wine and table grapes and has long been a subject of cultural and scientific interest. Flavour and aroma wheels are popular tools in evaluation the flavour and aroma profiles of various foods and beverages, including wines, for which they provide a visual representation of the most common aroma [4]. This allows producers, distributers, sellers, and consumers of wine to evaluate its quality using a shared language. In contrast, the flavour of table grapes is not commonly evaluated using flavour and aroma wheels, likely because aroma is less important than other characteristics.

Many grape cultivars are classified based on their flavour profile, such as muscat, foxy, muscadine, and neutral (no or weak flavour). The flavours of table grapes can be found in the literature and various databases [5–14]. Information on the flavour characteristics of most table grape cultivars is limited, with the exceptions of muscat and foxy; however, hybrid grapes have a variety of flavours that are difficult to express only in terms of muscat and foxy. Additionally, technical terms such as foxy and muscat may be unfamiliar to general consumers.

Aroma derives mainly from complex combinations of volatile compounds [3]; thus, the flavour of grapes is influenced by the composition and concentrations of volatile compounds, which depend on several factors, including genetics, species, location, climate, cultivation practices, and ripeness [15, 16]. The volatile compounds found in grapes include monoterpenes, C_13_-norisoprenoids, alcohols, esters, and carbonyls [17]. Esters and terpenes provide fruity and floral aroma characteristics [15], while the green leafy aroma of grapes is derived from C_6_-aldehydes and -alcohols [18]. Grape berries of the muscat cultivar (*V. vinifera* L.) exhibit a distinctive aroma associated with their high levels of monoterpenes [19]. Similarly, *V. labrusca*-derived hybrids and *V. rotundifolia* often contain methyl anthranilate, 2-aminoacetophenone, and furaneol, which contribute to their foxy aroma [20, 21]. Although distinctive flavour compounds of certain grape species have been identified, the flavour compounds of hybrid grape cultivars are poorly understood.

The volatile compound profiles of several table grapes have been identified using headspace-solid-phase microextraction (HS-SPME) [9, 16, 22–24]; however, this technique may be unable to identify volatile compounds with relatively high boiling points and low volatilities, which can contribute significantly to the overall aroma of table grapes. Solvent-assisted flavour evaporation (SAFE) is widely used to separate volatile compounds from complex matrices, and has proven effective in recovering trace compounds and high-boiling-point compounds at low temperatures [25–27]. SAFE has been applied in the analysis of volatiles in horticultural crops since its development in 1999, but its use in the analysis of the volatiles in grapes is limited.

**Table 1:** Variety and flavour characteristics of 38 table grape cultivars based on previous reports and databases

**Cultivars**	**Code** ^**a**^	**Varieties** ^**b**^	**Parents** ^**c**^	**Flavour type** ^**d**^	**References** ^**e**^
Muscat of Alexandria	MA	*V. vinifera*	Heptakilo × Muscat a Petits Grains	Muscat	5,7
Muscat Hamburg	MH	*V. vinifera*	Schiava Grossa × Muscat of Alexandria	Muscat	5,7,9
Neo Muscat	NE	*V. vinifera*	Muscat of Alexandria × Koshu Sanjaku	Muscat	5,6,7
Shine Muscat	SM	Hybrids between *V. vinifera* and *V. labrusca*	Akitsu 21 × Hakunan	Muscat	5,6,7
Hakunan	HA	*V. vinifera*	Katta Kurgan × Kaiji	Muscat	5,7
Muscat a Petis Grains Rouge	MPGR	*V. vinifera*	Muscat a Petits Grains Blancs Mutation	Muscat	5
Muscat a Petis Grains Blancs	MPGB	*V. vinifera*	Unknown	Muscat	5
Benitaka	BE	*V. vinifera*	Italia Mutation	Muscat	5
Katta Kurgan	KK	*V. vinifera*	Unknown	None	5,7
Rizamat	RI	*V. vinifera*	Katta Kurgan × Parkentskii	None	5
Alphonse Lavallee	AL	*V. vinifera*	Karistvala Kolkhuri × Muscat Hamburg	None, other	5,7
Rosaki	RO	*V. vinifera*	Unknown	None	5
Parkent	PA	*V. vinifera*	Unknown	None	5
Koshu	KO	*Vitis interspecific crossing*	Unknown	None	5,7
Buffalo	BU	*Vitis interspecific crossing*	Herbert × Watkins	Foxy	5,7
Sunny Rouge	SR	Hybrids between *V. vinifera, V. labrusca,* and *V. aestivalis*	Pione × Red Pearl	Foxy or none	5,7,10
Delaware	DE	Hybrids between *V. vinifera, V. labrusca,* and *V. aestivalis*	Unknown	Foxy, none	5,7
Muscat Bailey A	MBA	Hybrids between *V. vinifera, V. labrusca*, and *V. lincecomii*	Bailey × Muscat Hamburg	None, specific foxy	5,6,7
Oriental Star	OS	Hybrids between *V. vinifera* and *V. labrusca*	Akitsu 21 × Ruby Okuyama	None, other	5,6,7
Yuhou	YU	Hybrids between *V. vinifera* and *V. labrusca*	Shine Muscat × Tenzan	-	7
Steuben	ST	Hybrids between *V. vinifera* and *V. labrusca*	Wayne × Sheridan	Other	5,7
Keuka	KE	Hybrids between *V. vinifera* and *V. labrusca*	Chasselas Rose × Mills	Foxy, other	5,7
Honey Venus	HV	Hybrids between *V. vinifera* and *V. labrusca*	Benizuiho × Olympia	Other	5,8
Himrod	HI	Hybrids between *V. vinifera* and *V. labrusca*	Sultanina × Ontario	Other	5,7
Sun Verde	SV	Hybrids between *V. vinifera* and *V. labrusca*	Dark Ridge × Centennial	Other	5,7,11
Nagano Purple	NP	Hybrids between *V. vinifera* and *V. labrusca*	Kyoho × Rizamat	Foxy, favourable foxy	5,6
Kyoho	KY	Hybrids between *V. vinifera* and *V. labrusca*	Centennial × Ishihara Wase	Foxy, favourable foxy, strawberry	5,6,7,9
Aki Queen	AQ	Hybrids between *V. vinifera* and *V. labrusca*	Kyoho (4 N) × Kyoho (4 N)	Foxy, favourable foxy	5,6,7
Queen Nina	QN	Hybrids between *V. vinifera* and *V. labrusca*	Akitsu 20 × Aki Queen	None, foxy	5,7,13
Pione	PI	Hybrids between *V. vinifera* and *V. labrusca*	Kyoho × Muscat Cannon Hall (4 N)	Foxy	5,6,7
Fujiminori	FU	Hybrids between *V. vinifera* and *V. labrusca*	Ikawa Selection 682 × Pione	Foxy, distinct foxy, strawberry	5,9
Black Beet	BB	Hybrids between *V. vinifera* and *V. labrusca*	Fujiminori × Pione	None	9,12
Campbell Early	CE	Hybrids between *V. vinifera* and *V. labrusca*	Moore Early × (Belvidere × Muscat Hamburg)	Foxy	5,7
Niagara	NI	Hybrids between *V. vinifera* and *V. labrusca*	Concord × Cassady	Foxy	5,7,9
Concord	CO	Hybrids between *V. vinifera* and *V. labrusca*	Catawba × *V. labrusca* Linne	Foxy	5,7
Triumph	TR	*V. rotundifolia*	Fry × Georgia 29–49	Other, muscadine	5,7,14
Carlos	CA	*V. rotundifolia*	Howard × NC 11–173 (Topsail 10 Tarheel)	Muscadine	5,14
Fry	FR	*V. rotundifolia*	Georgia 19–93 × USDA 19–11	Muscadine	5,14

a Codes based on abbreviated cultivar names are used afterwards in the manuscript. The varieties ^b^, parents ^c^, and flavour types ^d^ are taken from the literature.

**Figure 1:**
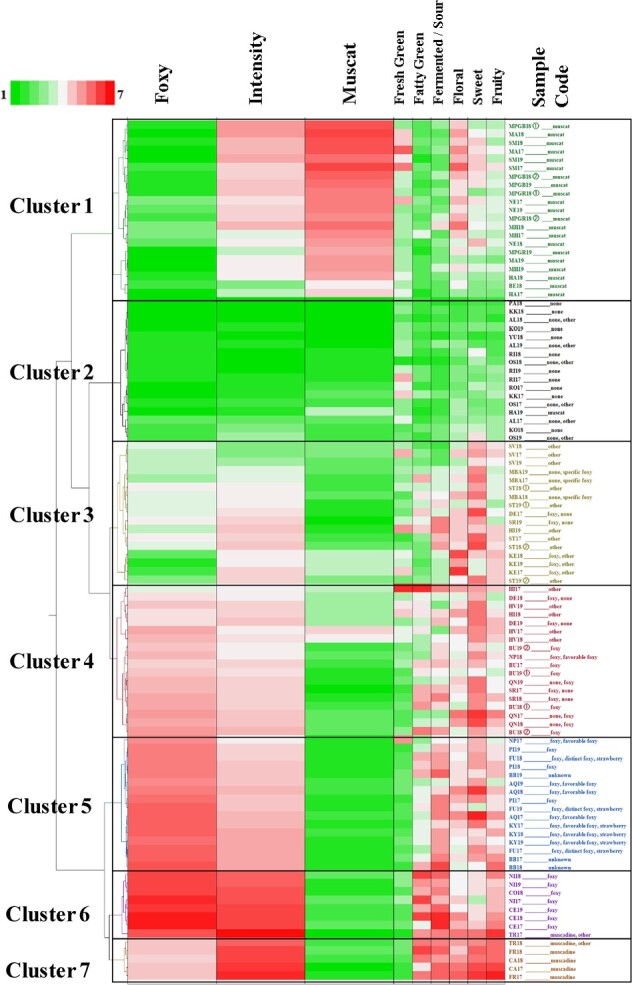
Classification of flavour characteristics by hierarchical cluster analysis (HCA) using Ward’s cluster algorithm for the dataset of flavour intensity values and foxy and muscat flavours obtained from the sensory evaluation of 102 samples. The heat maps indicate the mean sensory evaluation values of the nine types of flavour (n = 4). The samples are indicated by a code of abbreviated cultivar name (Table 1) followed by harvest year (*e.g.,* FR17 = Fry harvested in 2017). Table 1 gives the reference flavour type. ① and ② denote samples harvested from different trees in the same year. Lower (higher) sensory evaluation scores in the heat map are presented in green (red), as shown on the scale.

Both orthonasal olfaction (perceiving odours through the nostrils) and retronasal olfaction (perceiving odours through the throat while eating or drinking) are crucial in perceiving flavour. Odour activity values (OAVs; defined as the ratio of concentration to odour threshold) and gas chromatography-olfactometry (GC-O) are common screening tools to evaluate the potential importance of aroma compounds in foods. Several studies have used GC-O and OAVs to identify important flavours in table grapes [21–23]; however, most of these studies were based solely on orthonasal olfaction. In recent years, integrated analysis of large-scale sensory and chemical data, such as multivariate analysis, has enabled the identification of flavour- and taste-related volatiles. This provides novel insights into flavour chemistry, the interactions between taste and retronasal olfaction, and establishes a paradigm for enhancing the appeal of natural products [28].

Existing studies on the flavour and flavour compounds of table grapes have primarily examined foxy and muscat flavours, along with limited volatile component analysis and screening methods. Knowledge of other flavours and the compounds that contribute to them is comparatively lacking. In this study, we conducted sensory analysis to evaluate the flavour profiles of 38 table grape cultivars, including muscadine (*V. rotundifolia*). Volatile compounds in the grape berries were analysed using a combination of SAFE and gas chromatography–mass spectrometry (GC–MS). The relationship between the flavour and volatile compound profiles was examined using multivariate data analysis to identify the volatile compounds that contribute to grape flavours.

## Results

### Sensory characterisation

The flavour characteristics of hybrid table grapes were evaluated using a flavour wheel (Fig. S1), which was then used to investigate the flavour characteristics of a total of 102 samples from 38 table grape cultivars with various flavour types harvested over three years (Table 1, Table S1). Hierarchical cluster analysis (HCA) of the sensory evaluation data from the flavour intensity, foxy flavour and muscat flavour of 102 samples was applied to classify their flavour characteristics and categorise the samples accordingly (Fig. 1). Furthermore, fresh green, fatty green, fermented/sour, floral, sweet, and fruity flavours were also represented by heat maps (Fig. 1). The mean sensory evaluation scores of the clusters shown in Fig. 1 are listed in Table S2. The codes in Table 1 representing the abbreviated names of the cultivars are hereafter used throughout the manuscript.

Cluster analysis was carried out on the sensory evaluation data of 38 grape cultivars for the conventional elements of flavour classification, including foxy flavour, muscat flavour, and flavour intensity. The 102 samples were categorised into seven clusters (Fig. 1). Six cultivars (HA, HI, DE, SR, NP, and TR) occasionally clustered differently, whereas 27 cultivars displayed similar clustering across the years (Fig. 1). All samples in cluster 1 are of the muscat flavour type (Table 1). The grapes in this cluster displayed muscat, floral, fresh green, and sweet flavours with flavour intensities of at least (Table S2). With the exception of HA19, the samples in cluster 2 have a neutral flavour (Table 1). Indeed, cluster 2 showed the lowest flavour intensities among the clusters, with a fresh green value of 2.66, but no characteristic flavour (Table S2). Clusters 3 and 4 included a mixture of cultivars previously classified as having a foxy flavour or other flavour types (Table 1). Samples HV17 and HV18 exhibited mixed foxy and muscat flavours (Fig. 1). Cluster 3 exhibited sweet, floral, and fruity flavours with a high flavour intensity above three (Table S2). Cluster 4 was characterised by sweet, foxy, fruity, floral, fatty green, and fermented/sour flavours with a flavour intensity greater than three (Table S2). Clusters 5 and 6 were characterised by foxy, fruity, fermented/sour, fatty green, floral, and sweet flavour characteristics with a flavour intensity greater than three (Table S2). Cultivars in clusters 5 and 6 (with the exceptions of BB and TR17) have a foxy flavour (Table 1); however, cluster 5 showed considerably lower values in foxy, fatty green, and fermented/sour flavours than cluster 6 (Table S2). All samples in cluster 7 have muscadine flavours (Table 1). Although clusters 6 and 7 had similar flavour characteristics (Fig. 1), the intensity of the foxy flavour was lower in cluster 7 than in cluster 6 (which had the highest value of 6.25 for foxy flavour). Conversely, cluster 7 had a higher value for sweet flavour compared to cluster 6 (5.00 *vs*. 3.56).

### Analysis of volatile compounds by the SAFE method and GC–MS

Following SAFE extraction, several hundred peaks were detected in the GC–MS data, and 98 volatile compounds were identified (Table 2).

Of the 98 volatile compounds, 11 volatiles found in >90% of all samples (Table 2). These include three C_6_ compounds (hexanal, (*E*)-2-hexenal, and 3-hexenol), five acids (acetic acid, hexanoic acid, octanoic acid, nonanoic acid, and benzoic acid), one alcohol (benzyl alcohol), and two aldehydes (benzaldehyde and vanillin). The interquartile range (IQR) of the above compounds in all samples ranged from 7.36 μg·kg^−1^ fresh weight (FW) to 95.7 μg·kg^−1^ FW, representing nonanoic acid and (*E*)-2-hexenal, respectively (Table 2). In contrast, 74 compounds were identified with a detection frequency of less than 50.0% in all 102 samples (Table 2). Among them, esters (31 samples) and monoterpenoids (24 samples) were the most abundant. Ethyl 3-hydroxybutanoate, ethyl butanoate, ethyl 2-butenate, and ethyl hexanoate were the most abundant esters, while linalool, geraniol, nerol, and α-terpineol, were the most abundant monoterpenoids. Other esters detected included butyl acetate, hexyl acetate, hexyl hexanoate, butyl octanoate, benzyl acetate, β-phenethyl acetate, hexyl octanoate, phenethyl hexanoate, and phenylethyl octanoate*.* Additionally, slightly volatile water-soluble compounds with intermediate molecular weights were identified, including esters (*e.g.,* ethyl octanoate, ethyl decanoate, ethyl (*E,Z*)-2,4-decadienoate, and ethyl cinnamate), acids (*e.g.,* octanoic acid, nonanoic acid, decanoic acid, and benzoic acid), hydroxyl esters (*e.g.,* methyl 3-hydroxybutanoate, ethyl 3-hydroxybutanoate, and ethyl 3-hydroxyhexanoate), hydroxy monoterpenoids (8-hydroxylinalool), and polyols (*e.g.,* 2,6-dimethyl-1,7-octadien-3,6-diol). Furaneol, methyl anthranilate, methyl *N*-formylanthranilate, acetoin, and vanillin were also detected. The IQR among all compounds ranged from 0.189 μg·kg^−1^ FW (methyl salicylate) to 591 μg·kg^−1^ FW (phenylethyl alcohol), as shown in Table 2.

### Profiling of volatile compounds by principal component analysis

**Figure 2:**
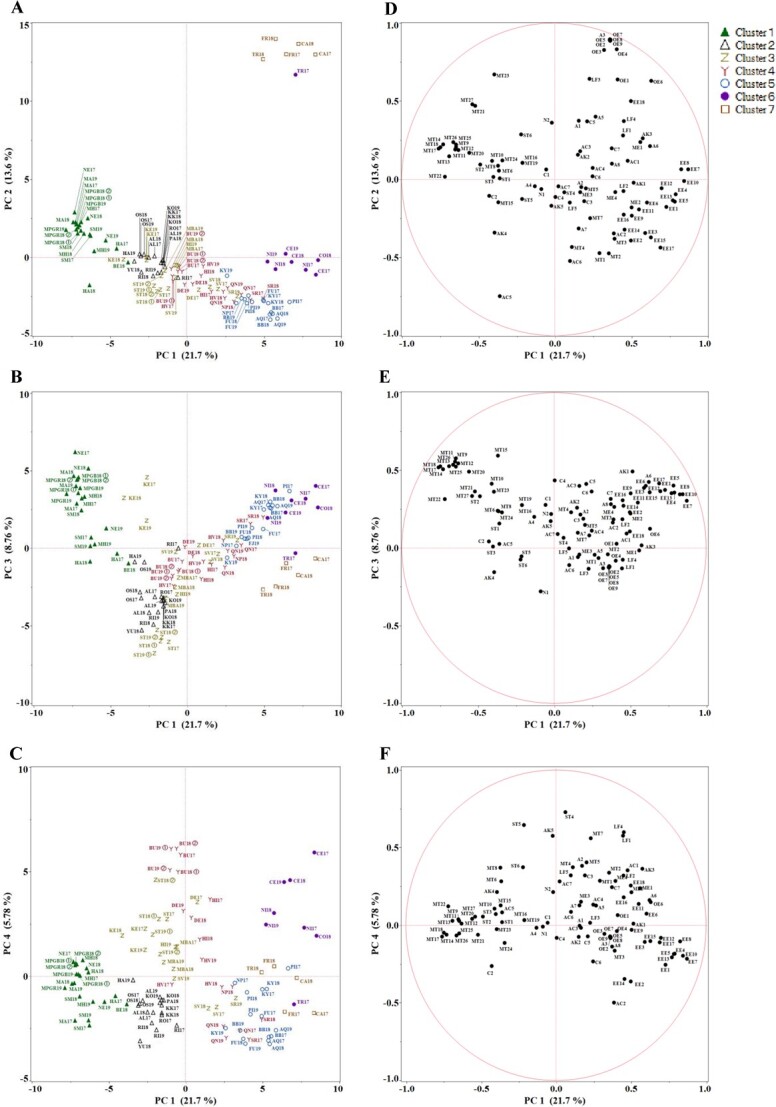
PCA of volatile compounds obtained from 102 grape samples. The colour of each sample represents its classification based on the flavour characteristics shown in Fig. 1. 2A–2C: Scatter plots of the PCA scores of all the samples. 2D–2F: The corresponding loading plots indicating the relative importance of the variables. Sample codes: cultivar, harvest year, and reference flavour (Table 1). Samples denoted ① and ② were harvested from different trees in the same year. Compound code: compound type and compound number (Table 2).

The log-transformed quantitative data obtained from the 98 volatile compounds in 102 fresh table grape samples were analysed using principal component analysis (PCA). The first four principal components accounted for 49.8% of the total variation in the data (Fig. 2).

**Table 2:** Variation in volatile compounds within table grape cultivars

**Codes**	**Compounds**	**RI** ^**a**^	**ID** ^**b**^	**Content μg·kg** ^**−1**^ **FW (range)**^**c**^	**IQR** ^**d**^	**DFR** ^**e**^
	C_6_ Compounds					
C1	Hexanal	1075	A	24.9 (0–193)	20.1	97.1
C2	3-hexenal	1134	A	132 (0–892)	181	86.3
C3	(*Z*)-2-hexenal	1189	B	0.488 (0–7.57)	-	17.6
C4	(*E*)-2-hexenal	1202	A	102 (0–870)	95.7	92.2
C5	1-hexanol	1350	A	49.2 (0–1170)	14.2	75.5
C6	3-hexenol	1377	A	32.1 (0–183)	30.4	92.2
C7	(*E*)-2-hexenol	1399	A	29.3 (0–1390)	12.8	70.6
	Alcohols and phenol					
A1	isoamyl alcohol	1189	A	0.155 (0–15.9)	-	0.98
A2	2,3-butanediol (1)	1531	A	30.0 (0–2340)	-	12.7
A3	octanol	1553	B	12.0 (0–372)	-	5.88
A4	2,3-butanediol (2)	1570	A	15.7 (0–419)	-	15.7
A5	Benzyl alcohol	1854	B	86.5 (0–1860)	46.2	99.0
A6	Phenylethyl alcohol	1888	A	2510 (0–62 400)	591	83.3
A7	1-dodecanol	1962	B	19.0 (0–288)	5.03	46.1
A8	Phenol	1984	B	4.97 (0–76.4)	5.05	70.6
	Methyl esters					
ME1	Methyl 3-hydroxybutanoate	1465	A	13.2 (0–321)	4.23	29.4
ME2	Methyl salicylate	1738	A	2.08 (0–59.1)	0.189	25.5
ME3	Methyl anthranilate	2198	A	0.173 (0–15.9)	-	3.92
ME4	Methyl *N*-formylanthranilate	2518	A	1.10 (0–66.3)	-	7.84
	Ethyl esters					
EE1	Ethyl butanoate	1030	A	58.0 (0–694)	80.4	41.2
EE2	Ethyl 2-methylbutanoate	1049	B	4.58 (0–66.6)	-	22.5
EE3	Ethyl pentanoate	1130	A	1.74 (0–20.7)	0.385	25.5
EE4	Ethyl 2-butenoate	1153	A	27.3 (0–313)	28.8	39.2
EE5	Ethyl hexanoate	1226	A	29.3 (0–277)	31.3	41.2
EE6	Ethyl heptanoate	1326	A	0.884 (0–12.3)	-	16.7
EE7	Ethyl octanoate	1423	A	16.9 (0–191)	14.5	38.2
EE8	Ethyl 3-hydroxybutanoate	1503	A	167 (0–1650)	230	43.1
EE9	Ethyl nonanoate	1524	A	0.418 (0–8.13)	-	12.7
EE10	Ethyl decanoate	1626	A	22.2 (0–291)	19.2	39.2
EE11	Ethyl benzoate	1637	A	5.33 (0–282)	-	20.6
EE12	Ethyl trans-4-decenoate	1652	A	5.73 (0–98.3)	-	23.5
EE13	Ethyl 3-hydroxyhexanoate	1664	A	8.85 (0–230)	8.06	32.4
EE14	Ethyl trans-2-decenoate	1744	B	0.668 (0–17.8)	-	14.7
EE15	Ethyl benzeneacetate	1761	A	15.4 (0–375)	4.34	28.4
EE16	Ethyl salicylate	1775	B	3.17 (0–104)	-	16.7
EE17	Ethyl (*E,Z*)-2,4-decadienoate	1823	A	115 (0–5110)	27.2	38.2
EE18	ethyl cinnamate	2090	A	2.20 (0–105)	-	9.80
	Other Esters					
OE1	Butyl acetate	1069	A	8.90 (0–463)	-	6.86
OE2	Hexyl acetate	1265	A	3.86 (0–166)	-	5.88
OE3	Hexyl hexanoate	1597	A	2.91 (0–143)	-	4.90
OE4	Butyl octanoate	1600	A	4.98 (0–215)	-	6.86
OE5	Benzyl acetate	1702	A	6.69 (0–424)	-	5.88
OE6	Β-phenethyl acetate	1788	A	555 (0–18 300)	-	18.6
OE7	Hexyl octanoate	1797	A	4.87 (0–177)	-	5.88
OE8	Phenethyl hexanoate	2144	A	1160 (0–45 800)	-	5.88
OE9	Phenylethyl octanoate	2361	A	749 (0–32 700)	-	5.88
	Acids					
AC1	Acetic acid	1439	A	59.7 (0–570)	48.4	96.1
AC2	Butanoic acid	1612	B	1.26 (0–41.9)	-	18.6
AC3	Hexanoic acid	1830	A	23.1 (0–226)	14.9	91.2
AC4	Octanoic acid	2048	B	17.6 (0–719)	8.14	90.2
AC5	Nonanoic acid	2154	B	29.9 (0–1520)	7.36	93.1
AC6	Decanoic acid	2262	B	18.5 (0–996)	2.15	69.6
AC7	Benzoic acid	2417	B	182 (0–5390)	57.9	97.1
	Aldehydes and ketones					
AK1	Acetoin	1268	A	269 (0–2180)	458	84.3
AK2	Benzaldehyde	1491	A	15.1 (0–191)	14.4	91.2
AK3	Phenylacetaldehyde	1610	A	28.4 (0–492)	21.3	36.3
AK4	Vanillin	2531	A	62.1 (0–1970)	30.7	96.1
AK5	Methyl vanillate	2583	A	5.83 (0–198)	3.09	40.2
	Lactones and furanones					
LF1	Mesifurane	1570	A	175 (0–8380)	2.16	26.5
LF2	γ-butyrolactone	1589	B	11.7 (0–216)	12.5	50.0
LF3	γ-hexalactone	1664	A	2.08 (0–164)	-	2.94
LF4	furaneol	2015	A	92.8 (0–3260)	2.30	30.4
LF5	γ-decanolactone	2106	A	0.629 (0–12.4)	-	7.84
	Monoterpenoids					
MT1	α-pinene	1017	A	32.5 (0–846)	5.22	46.1
MT2	β-pinene	1096	B	31.6 (0–640)	6.48	45.1
MT3	β-phellandrene	1111	C	0.393 (0–5.25)	-	22.5
MT4	Limonene	1182	B	10.8 (0–416)	3.45	64.7
MT5	Eucalyptol	1192	B	0.423 (0–14.4)	-	5.88
MT6	(*Z*)-β-ocimene	1228	B	0.561 (0–10.5)	-	16.7
MT7	γ-terpinene	1230	B	0.882 (0–13)	0.225	26.5
MT8	(*E*)-β-ocimene	1240	B	0.845 (0–18.5)	-	12.7
MT9	*cis*-rose oxide	1338	A	1.37 (0–22.3)	-	17.6
MT10	*trans*-roseoxide	1350	A	0.428 (0–21.6)	-	8.82
MT11	*trans*-linalooloxide (furanoid)	1427	A	5.17 (0–220)	-	22.5
MT12	*cis*-linalooloxide (furanoid)	1455	A	4.72 (0–115)	-	21.6
MT13	Linalool	1541	A	97.0 (0–2360)	10.3	35.3
MT14	Hotrienol	1600	B	19.2 (0–381)	-	24.5
MT15	α-terpineol	1680	A	43.0 (0–1320)	15.4	61.8
MT16	α-citral	1715	A	0.0629 (0–5.13)	-	1.96
MT17	*trans*-linalooloxide (pyranoid)	1722	A	28.0 (0–830)	1.14	26.5
MT18	*cis*-linalooloxide (pyranoid)	1751	A	21.6 (0–1020)	-	23.5
MT19	β-citronellol	1759	A	0.529 (0–48.6)	-	1.96
MT20	Nerol	1788	A	3.00 (0–64.6)	-	15.7
MT21	Guaniol	1838	A	8.23 (0–149)	1.74	26.5
MT22	2,6-dimethyl-3,7-octadiene-2,6-diol	1943	B	382 (0–11 600)	85.8	52.9
MT23	6,7-dihydro-7-hydroxylinalool	1976	B	11.3 (0–548)	1.12	25.5
MT24	3,7-dimethyl-1, 7-octadien-6-ol	2018	C	0.184 (0–8.32)	-	4.90
MT25	2,6-dimethyl-1,7-octadiene-3,6-diol	2123	B	7.30 (0–318)	-	16.7
MT26	8-hydroxylinalool	2268	B	5.45 (0–216)	-	16.7
MT27	Geranic acid	2327	A	34.6 (0–2000)	4.09	29.4
	Sesquiterpenes					
ST1	β-caryophyllene	1567	A	17.5 (0–1460)	-	17.6
ST2	α-caryophyllene	1636	B	61.9 (0–4680)	-	12.7
ST3	(*Z*)-β-farnesene	1652	B	134 (0–13 000)	-	22.5
ST4	α-farnesene (1)	1712	B	123 (0–12 200)	-	20.6
ST5	α-farnesene (2)	1732	B	512 (0–47 100)	8.61	46.1
ST6	Calamenene	1799	B	82.1 (0–1830)	55.3	88.2
	C₁₃ Norisoprenoids					
N1	β-damascenone	1791	A	1.17 (0–35.2)	-	9.80
N2	β-ionone	1906	A	6.83 (0–100)	6.25	74.5

a Retention indices were determined using a DB‐WAX UI column.

b Identification of volatile compounds: A, MS and RI were consistent with standards; B, MS and RI were consistent with MS database and literature data; C, identified by MS and RI agreed with MS database.

c Mean volatile concentrations (μg·kg^−1^ FW) and their distribution ranges (in parentheses) in grape berries.

d Interquartile range (μg·kg^−1^ FW).‘-’ indicates that the majority of compounds were not detected in the samples and have a value of zero.

e Detection frequency of compounds in all samples (%).

**Figure 3:**
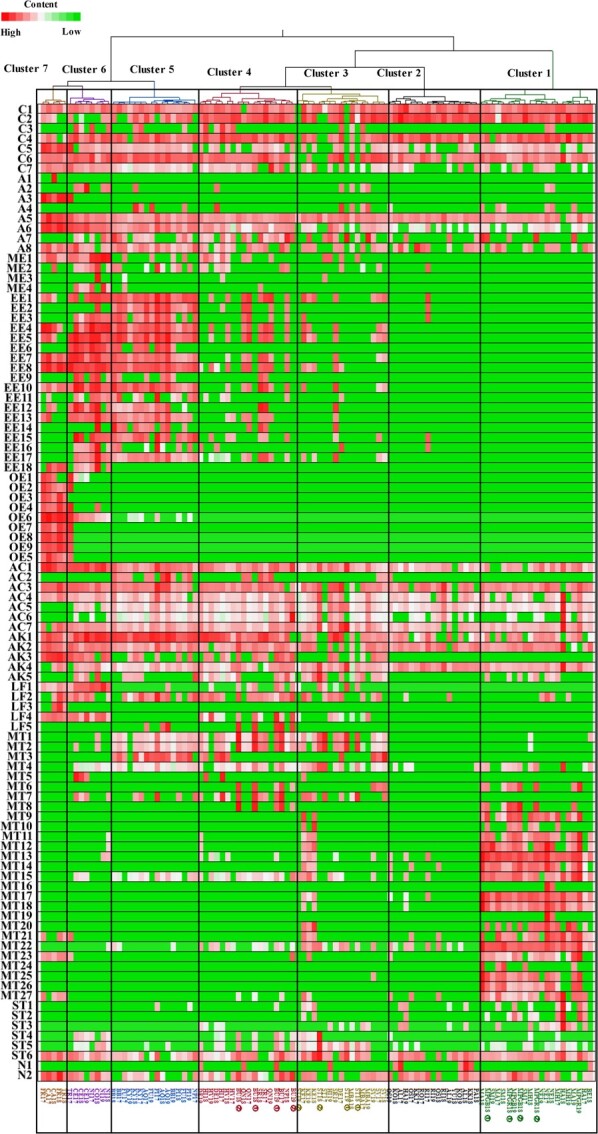
Cluster analysis of 102 grape samples and quantified volatile compounds. The contents of 98 volatile compounds were quantified as 3-heptanol equivalents, log-transformed, and plotted in a heat map, in which lower (higher) volatile compound contents are presented in green (red), as shown on the scale. The samples are indicated by a code of abbreviated cultivar name (Table 1) followed by harvest year (*e.g.,* FR17 = Fry harvested in 2017). ① and ② denote samples harvested from different trees in the same year. The cluster classification of the samples is based on the classification by sensory evaluation in Fig. 1.

In the plot plane, the samples fell into the same clusters identified in the sensory-sorting task. PC1 shows that monoterpenoids contributed to cluster 1, and ethyl esters to clusters 5–7 (Fig. 2A and 2D), thereby explaining the separation of cluster 1 from clusters 5, 6, and 7. PC2 explained the separation of cluster 7 from the other clusters; Hexyl, butyl, and phenylethyl esters and their corresponding alcohols contributed to the formation of other clusters, but not cluster 7 (Fig. 2A and 2D). Although clusters 2, 3, and 4 overlapped in PC1 and PC2 (Fig. 2A), the volatile compound profiles of these clusters were characterised by PC3 and PC4 (Fig. 2). PC1 and PC3 indicated the presence of mixed ethyl esters and monoterpenoids in cultivars such as ‘Keuka’ (Fig. 2B and 2E). PC1 and PC4 separated clusters 3, 4, and 6. The samples in these clusters contained several compounds such as farnesene, furaneol, and mesifurane (Fig. 2C and 2F).

### Content of volatile compounds for each grape flavour classification based on sensory evaluation

The content of volatile compounds in each grape flavour classification based on Fig 1 is represented in the heat map (Fig. 3), and the content of volatile compounds in each cluster is summarised in Table 3. No significant difference was observed in the total content of clusters 1–6 (*p* > 0.05), with cluster 2 having the lowest total volatile content, and cluster 6 having the highest (Table 3). Cluster 1 had a higher monoterpenoid content than the other clusters, while cluster 2 was dominated by C_6_ compounds (Table 3). Clusters 3 and 4 tended to have the same volatile profile as cluster 2, but typically contained more ethyl esters, alcohols, monoterpenoids, lactones, and furanones (Table 3). Cluster 3 contained more sesquiterpenes, while cluster 4 contained more lactones and furanones such as furaneol and γ-decanolactone (Table 3). Cluster 5 was characterised by abundant alcohols, ethyl esters, and aldehydes and ketones (Table 3). Cluster 6 was dominated by alcohols, ethyl esters, lactones and furanones, and methyl esters (Table 3). Conversely, the total volatile compound content in cluster 7 was higher than that of all the other clusters (Table 3). Cluster 7 was dominated by esters, including ethyl esters and alcohols such as butyl, hexyl, and phenethyl alcohol (Table 3). The content of these classes of compounds is significantly higher than that of other clusters, including C_13_ norisoprenoids (Table 3).

**Table 3:** Concentrations (μg·kg^−1^ FW) of volatile compounds between clusters determined based on Fig. 1

**Codes**	**Compounds**	**Cluster 1**		**Cluster 2**		**Cluster 3**		**Cluster 4**		**Cluster 5**		**Cluster 6**		**Cluster 7**	
**C1**	**hexanal**	25.5 ± 3.40	^A^	40.9 ± 11.6	^A^	24.6 ± 6.13	^A^	21.9 ± 4.04	^A^	16.1 ± 3.70	^A^	19.9 ± 3.86	^A^	16.4 ± 5.32	^A^
**C2**	**3-hexenal**	181 ± 410	^AB^	230 ± 47.1	^A^	149 ± 32.5	^AB^	137 ± 26.2	^AB^	44.3 ± 13.3	^B^	6.79 ± 3.53	^B^	5.49 ± 3.07	^B^
**C3**	**(*Z*)-2-hexenal**	0.124 ± 0.0871	^AB^	-	^B^	0.435 ± 0.280	^AB^	1.08 ± 0.529	^AB^	0.362 ± 0.245	^AB^	1.81 ± 0.927	^A^	-	^AB^
**C4**	**(*E*)-2-hexenal**	138 ± 40.0	^A^	116 ± 25.0	^A^	48.4 ± 14.8	^A^	126 ± 25.2	^A^	84.7 ± 19.1	^A^	121 ± 33.8	^A^	18.1 ± 10.3	^A^
**C5**	**1-hexanol**	8.78 ± 1.50	^B^	6.05 ± 1.85	^B^	8.58 ± 2.61	^B^	8.94 ± 2.27	^B^	12.1 ± 1.51	^B^	163 ± 144	^B^	585 ± 151	^A^
**C6**	**3-hexenol**	25.6 ± 8.27	^A^	17.2 ± 3.96	^A^	38.7 ± 10.8	^A^	31.4 ± 7.74	^A^	48.3 ± 5.47	^A^	45.5 ± 13.9	^A^	17.8 ± 2.60	^A^
**C7**	**(*E*)-2-hexenol**	5.53 ± 1.82	^A^	8.38 ± 3.93	^A^	12.5 ± 7.38	^A^	98.9 ± 76.4	^A^	5.56 ± 0.909	^A^	46.4 ± 21.6	^A^	55.9 ± 10.9	^A^
	**C**_**6**_ **Compounds Subtotal**	25.6 ± 8.27	^AB^	418 ± 75.0	^AB^	282 ± 60.7	^AB^	425 ± 82.3	^AB^	212 ± 29.3	^B^	404 ± 171	^AB^	699 ± 156	^A^
	**C**_**6**_ **Compounds Subtotal (%)**	7.18		48.6		4.51		16.2		5.99		2.40		0.834	
**A1**	**isoamyl alcohol**	-	^B^	-	^B^	-	^B^	-	^B^	-	^B^	-	^B^	3.17 ± 3.17	^A^
**A2**	**2,3-butanediol (1)**	1.65 ± 1.22	^B^	-	^B^	9.30 ± 5.05	^B^	6.00 ± 6.00	^B^	9.84 ± 9.84	^B^	326 ± 289	^A^	-	^AB^
**A3**	**octanol**	-	^B^	-	^B^	-	^B^	-	^B^	-	^B^	28.1 ± 28.1	^B^	200 ± 56.2	^A^
**A4**	**2,3-butanediol (2)**	4.92 ± 1.98	^A^	0.167 ± 0.167	^A^	26.6 ± 18.5	^A^	16.0 ± 16.0	^A^	46.8 ± 30.1	^A^	-	^A^	-	^A^
**A5**	**benzyl alcohol**	44.2 ± 10.2	^B^	58.1 ± 14.3	^B^	67.2 ± 130	^B^	42.9 ± 5.90	^B^	52.8 ± 15.8	^B^	98.6 ± 34.3	^B^	672 ± 301	^A^
**A6**	**phenylethyl alcohol**	14.9 ± 4.00	^C^	22.9 ± 16.6	^C^	297 ± 215	^C^	326 ± 62.8	^C^	1350 ± 525	^BC^	6460 ± 2100	^B^	34 300 ± 8570	^A^
**A7**	**1-dodecanol**	26.1 ± 12.2	^A^	3.22 ± 1.89	^A^	41.5 ± 22.4	^A^	21.6 ± 10.8	^A^	11.8 ± 7.42	^A^	6.41 ± 4.28	^A^	-	^A^
**A8**	**phenol**	2.63 ± 0.809	^A^	8.08 ± 4.51	^A^	2.32 ± 0.591	^A^	3.88 ± 1.49	^A^	5.93 ± 1.36	^A^	6.80 ± 3.92	^A^	11.2 ± 4.13	^A^
	**Alcohols and Phenol Subtotal**	94.4 ± 17.7	^C^	92.4 ± 22.2	^C^	444 ± 217	^C^	416 ± 66.7	^C^	1470 ± 535	^BC^	6920 ± 2330	^B^	35 200 ± 8700	^A^
	**Alcohols and Phenol (%)**	1.77		10.7		7.11		15.9		41.7		41.1		42.0	
**ME1**	**methyl 3-hydroxybutanoate**	-	^B^	-	^B^	6.82 ± 3.33	^B^	5.19 ± 1.93	^B^	2.86 ± 1.49	^B^	123 ± 43.6	^A^	22.0 ± 10.4	^B^
**ME2**	**methyl salicylate**	-	^A^	1.02 ± 1.02	^A^	0.415 ± 0.286	^A^	1.36 ± 0.656	^A^	5.36 ± 3.65	^A^	7.13 ± 5.1	^A^	3.99 ± 3.99	^A^
**ME3**	**methyl anthranilate**	-	^B^	-	^B^	0.0736 ± 0.0736	^AB^	0.0236 ± 0.0236	^B^	0.00946 ± 0.00946	^AB^	1.98 ± 1.98	^A^	-	^AB^
**ME4**	**methyl *N*-formylanthranilate**	-	^B^	-	^B^	-	^B^	0.274 ± 0.274	^B^	0.201 ± 0.201	^B^	13.0 ± 8.07	^A^	-	^B^
	**Methyl Esters Subtotal**	-	^B^	1.02 ± 1.02	^B^	7.31 ± 3.41	^B^	6.85 ± 1.98	^B^	8.43 ± 3.71	^B^	145 ± 50.9	^A^	26.0 ± 13.0	^B^
	**Methyl Esters Subtotal (%)**	-		0.118		0.117		0.262		0.239		0.862		0.0311	
**EE1**	**ethyl butanoate**	-	^C^	14.0 ± 14.0	^C^	27.8 ± 12.2	^BC^	73.3 ± 29.1	^ABC^	163 ± 41.6	^A^	146 ± 29.4	^AB^	21.2 ± 13.0	^ABC^
**EE2**	**ethyl 2-methylbutanoate**	-	^B^	1.18 ± 1.18	^B^	-	^B^	4.84 ± 2.61	^B^	19.4 ± 4.98	^A^	6.20 ± 6.20	^AB^	-	^B^
**EE3**	**ethyl pentanoate**	-	^C^	0.669 ± 0.669	^BC^	0.513 ± 0.513	^C^	1.44 ± 0.849	^BC^	5.80 ± 1.46	^A^	4.83 ± 1.30	^AB^	-	^BC^
**EE4**	**ethyl 2-butenoate**	-	^C^	-	^C^	8.74 ± 4.39	^BC^	17.9 ± 6.22	^BC^	47.8 ± 9.46	^B^	117 ± 35.7	^A^	123 ± 61.3	^A^
**EE5**	**ethyl hexanoate**	-	^C^	-	^C^	8.35 ± 4.66	^C^	19.1 ± 8.01	^C^	80.1 ± 18.9	^B^	138 ± 31.4	^A^	23.3 ± 13.5	^BC^
**EE6**	**ethyl heptanoate**	-	^C^	-	^C^	-	^C^	-	^C^	2.36 ± 0.687	^B^	6.54 ± 1.24	^A^	-	^C^
**EE7**	**ethyl octanoate**	-	^C^	-	^C^	2.43 ± 2.11	^C^	10.0 ± 5.15	^C^	26.1 ± 5.89	^BC^	94.7 ± 24.3	^A^	65.4 ± 31.3	^AB^
**EE8**	**ethyl 3-hydroxybutanoate**	-	^C^	-	^C^	18.0 ± 10.3	^C^	93.1 ± 27.5	^BC^	264 ± 38.3	^B^	754 ± 154	^A^	955 ± 222	^A^
**EE9**	**ethyl nonanoate**	-	^B^	-	^B^	-	^B^	0.336 ± 0.243	^B^	0.726 ± 0.309	^B^	3.13 ± 1.22	^A^	-	^B^
**EE10**	**ethyl decanoate**	-	^C^	-	^C^	8.30 ± 6.36	^BC^	19.4 ± 11.1	^BC^	49.7 ± 11.6	^B^	112 ± 36.5	^A^	17.7 ± 4.78	^BC^
**EE11**	**ethyl benzoate**	-	^A^	-	^A^	0.298 ± 0.298	^A^	0.629 ± 0.384	^A^	26.6 ± 17.7	^A^	12.3 ± 5.03	^A^	0.691 ± 0.691	^A^
**EE12**	**ethyl trans-4-decenoate**	-	^B^	-	^B^	2.89 ± 2.89	^B^	9.51 ± 6.60	^B^	4.94 ± 2.03	^B^	34.1 ± 13.2	^A^	2.44 ± 1.70	^B^
**EE13**	**ethyl 3-hydroxyhexanoate**	-	^B^	-	^B^	0.618 ± 0.618	^B^	4.09 ± 1.45	^B^	18.1 ± 5.50	^B^	53.7 ± 25.4	^A^	19.9 ± 15.4	^AB^
**EE14**	**ethyl trans-2-decenoate**	-	^A^	-	^A^	0.297 ± 0.297	^A^	0.724 ± 0.442	^A^	2.02 ± 0.802	^A^	2.22 ± 2.22	^A^	-	^A^
**EE15**	**ethyl benzeneacetate**	-	^C^	2.58 ± 2.58	^BC^	0.872 ± 0.714	^BC^	3.02 ± 1.47	^BC^	45.2 ± 17.7	^AB^	91.6 ± 43.9	^A^	-	^BC^
**EE16**	**ethyl salicylate**	-	^A^	0.729 ± 0.729	^A^	2.02 ± 2.02	^A^	0.863 ± 0.609	^A^	8.54 ± 6.37	^A^	15.5 ± 12.7	^A^	-	^A^
**EE17**	**ethyl (*E,Z*)-2,4-decadienoate**	-	^B^	-	^B^	82.8 ± 76.9	^AB^	116 ± 70.6	^AB^	138 ± 49.4	^AB^	750 ± 624	^A^	-	^AB^
**EE18**	**ethyl cinnamate**	-	^B^	-	^B^	-	^B^	-	^B^	-	^B^	15.2 ± 12.8	^A^	20.6 ± 7.14	^A^
	**Ethyl Esters Subtotal**	-	^C^	19.1 ± 19.1	^C^	164 ± 107	^BC^	374 ± 140	^BC^	903 ± 189	^B^	2360 ± 780	^A^	1250 ± 301	^AB^
	**Ethyl Esters Subtotal (%)**	-		2.22		2.62		14.3		25.5		14.0		1.49	
**OE1**	**butyl acetate**	-	^B^	-	^B^	-	^B^	-	^B^	-	^B^	58.4 ± 57.8	^AB^	88.2 ± 50.1	^A^
**OE2**	**hexyl acetate**	-	^B^	-	^B^	-	^B^	-	^B^	-	^B^	20.8 ± 20.8	^AB^	45.5 ± 23.3	^A^
**OE3**	**hexyl hexanoate**	-	^B^	-	^B^	-	^B^	-	^B^	-	^B^	-	^B^	59.3 ± 23.9	^A^
**OE4**	**butyl octanoate**	-	^B^	-	^B^	-	^B^	-	^B^	-	^B^	31.1 ± 26.6	^A^	51.9 ± 22.0	^A^
**OE5**	**benzyl acetate**	-	^B^	-	^B^	-	^B^	-	^B^	-	^B^	7.41	^B^	125	^A^
**OE6**	**β-phenethyl acetate**	-	^B^	-	^B^	-	^B^	-	^B^	1.72 ± 1.26	^B^	923 ± 792	^B^	9840 ± 2330	^A^
**OE7**	**hexyl octanoate**	-	^B^	-	^B^	-	^B^	-	^B^	-	^B^	22.1 ± 22.1	^B^	64.0 ± 18.8	^A^
**OE8**	**phenethyl hexanoate**	-	^B^	-	^B^	-	^B^	-	^B^	-	^B^	1620 ± 1620	^B^	21 000 ± 7030	^A^
**OE9**	**phenylethyl octanoate**	-	^B^	-	^B^	-	^B^	-	^B^	-	^B^	954 ± 954	^B^	13 800 ± 4830	^A^
	**Other Ester Subtotal**	-	^B^	-	^B^	-	^B^	-	^B^	1.72 ± 1.26	^B^	3630 ± 3490	^B^	45 100 ± 12 600	^A^
	**Other Ester Subtotal (%)**	-		-		-		-		0.0487		21.6		53.8	
**AC1**	**acetic acid**	19.4 ± 6.04	^B^	7.84 ± 1.57	^B^	45.9 ± 25.4	^B^	49.7 ± 16.8	^B^	52.4 ± 20.1	^B^	210 ± 47	^A^	271 ± 95.4	^A^
**AC2**	**butanoic acid**	-	^B^	0.187 ± 0.187	^B^	0.146 ± 0.100	^B^	1.97 ± 0.882	^AB^	5.48 ± 2.62	^A^	-	^AB^	-	^AB^
**AC3**	**hexanoic acid**	15.7 ± 3.27	^A^	20.7 ± 9.03	^A^	26.2 ± 9.94	^A^	15.5 ± 2.16	^A^	33.2 ± 13.9	^A^	23.9 ± 4.86	^A^	45.0 ± 17.5	^A^
**AC4**	**octanoic acid**	40.0 ± 34.0	^A^	8.47 ± 3.77	^A^	16.6 ± 8.86	^A^	8.21 ± 2.09	^A^	14.1 ± 7.23	^A^	7.32 ± 1.69	^A^	19.4 ± 7.71	^A^
**AC5**	**nonanoic acid**	79.4 ± 71.8	^A^	10.7 ± 4.41	^A^	39.8 ± 14.5	^A^	16.8 ± 7.18	^A^	11.5 ± 2.97	^A^	5.34 ± 1.49	^A^	-	^A^
**AC6**	**decanoic acid**	28.5 ± 26.9	^A^	2.67 ± 1.59	^A^	8.54 ± 4.24	^A^	58.4 ± 55.2	^A^	2.84 ± 0.723	^A^	0.593 ± 0.306	^A^	-	^A^
**AC7**	**benzoic acid**	284 ± 244	^A^	24.0 ± 5.48	^A^	534 ± 325	^A^	56.4 ± 13.5	^A^	46.6 ± 9.02	^A^	67.1 ± 11.8	^A^	163 ± 72.9	^A^
	**Acids Subtotal**	467 ± 380	^A^	74.6 ± 19.1	^A^	671 ± 362	^A^	207 ± 85.8	^A^	166 ± 47.9	^A^	315 ± 55.2	^A^	499 ± 79.4	^A^
	**Acids Subtotal (%)**	8.74		8.67		10.7		7.90		4.70		1.87		0.595	
**AK1**	**acetoin**	30.0 ± 7.11	^D^	35.7 ± 14.9	^CD^	146 ± 81.2	^CD^	346 ± 79.1	^BC^	638 ± 90.3	^AB^	773 ± 240	^A^	227 ± 92.4	^BCD^
**AK2**	**benzaldehyde**	19.1 ± 8.79	^A^	14.6 ± 2.99	^A^	9.73 ± 2.19	^A^	12.8 ± 4.15	^A^	11.8 ± 3.67	^A^	19.8 ± 3.95	^A^	28.7 ± 8.49	^A^
**AK3**	**phenylacetaldehyde**	-	^C^	-	^C^	12.5 ± 5.41	^BC^	34.1 ± 11.1	^BC^	10.5 ± 4.69	^BC^	62.8 ± 16.8	^B^	280 ± 67.8	^A^
**AK4**	**vanillin**	117 ± 92.7	^A^	65.7 ± 21.3	^A^	107 ± 62.1	^A^	28.8 ± 7.00	^A^	11.8 ± 2.54	^A^	17.9 ± 8.34	^A^	19.4 ± 11.1	^A^
**AK5**	**methyl vanillate**	1.28 ± 0.436	^A^	-	^A^	16.3 ± 11.4	^A^	5.30 ± 2.48	^A^	5.86 ± 4.94	^A^	12.7 ± 9.96	^A^	-	^A^
	**Aldehydes and Ketones Subtotal**	167 ± 100	^C^	116 ± 25.6	^C^	291 ± 97.5	^BC^	428 ± 74.5	^ABC^	678 ± 91.8	^AB^	886 ± 236	^A^	555 ± 133	^ABC^
	**Aldehydes and Ketones Subtotal (%)**	3.13		13.5		4.66		16.3		19.2		5.26		0.663	
**LF1**	**mesifurane**	-	^B^	-	^B^	178 ± 146	^B^	35.1 ± 18.7	^B^	3.06 ± 3.06	^B^	1730 ± 969	^A^	52.8 ± 21.5	^B^
**LF2**	**γ-butyrolactone**	2.7.0 ± 1.90	^A^	1.10 ± 1.00	^A^	9.18 ± 3.51	^A^	17.6 ± 5.06	^A^	27.9 ± 13.6	^A^	11.4 ± 4.52	^A^	20.4 ± 15.4	^A^
**LF3**	**γ-hexalactone**	-	^B^	-	^B^	-	^B^	-	^B^	-	^B^	-	^B^	42.4 ± 31.4	^A^
**LF4**	**furaneol**	-	^A^	-	^A^	12.9 ± 9.69	^A^	317 ± 188	^A^	-	^A^	311 ± 138	^A^	209 ± 45.2	^A^
**LF5**	**γ-decanolactone**	-	^B^	-	^B^	-	^B^	2.62 ± 1.04	^A^	1.06 ± 0.786	^AB^	-	^AB^	-	^AB^
	**Lactones and Furanones Subtotal**	2.70 ± 1.90	^B^	1.10 ± 1.00	^B^	200 ± 145	^B^	373 ± 207	^B^	32.0 ± 14.1	^B^	2050 ± 1000	^A^	325 ± 80.2	^B^
	**Lactones and Furanones Subtotal (%)**	0.0505		0.128		3.20		14.2		0.906		12.2		0.388	
**MT1**	**α-pinene**	0.444 ± 0.369	^A^	-	^A^	80 ± 40.2	^A^	102 ± 52.9	^A^	6.61 ± 3.1	^A^	1.20 ± 1.20	^A^	-	^A^
**MT2**	**β-pinene**	0.0378 ± 0.0378	^A^	-	^A^	78.8 ± 36.1	^A^	96.8 ± 45.5	^A^	7.60 ± 3.37	^A^	1.93 ± 1.26	^A^	-	^A^
**MT3**	**β-phellandrene**	-	^B^	-	^B^	0.645 ± 0.346	^AB^	0.467 ± 0.233	^AB^	1.29 ± 0.242	^A^	-	^B^	-	^B^
**MT4**	**limonene**	23.2 ± 19.7	^A^	0.519 ± 0.293	^A^	24.6 ± 13.4	^A^	7.63 ± 3.10	^A^	2.66 ± 0.885	^A^	1.10 ± 0.468	^A^	-	^A^
**MT5**	**eucalyptol**	-	^B^	-	^B^	0.0813 ± 0.0813	^B^	1.08 ± 0.83	^AB^	-	^B^	2.80 ± 1.83	^A^	-	^AB^
**MT6**	**(*Z*)-β-ocimene**	1.33 ± 0.574	^A^	-	^A^	0.416 ± 0.235	^A^	1.23 ± 0.635	^A^	-	^A^	-	^A^	-	^A^
**MT7**	**γ-terpinene**	-	^B^	-	^B^	1.39 ± 0.608	^AB^	2.57 ± 1.00	^A^	0.971 ± 0.628	^AB^	0.561 ± 0.292	^AB^	-	^AB^
**MT8**	**(*E*)-β-ocimene**	1.89 ± 0.779	^A^	-	^A^	-	^A^	2.58 ± 1.32	^A^	-	^A^	-	^A^	-	^A^
**MT9**	***cis*-rose oxide**	5.56 ± 1.34	^A^	-	^B^	1.38 ± 1.04	^B^	-	^B^	-	^B^	-	^B^	-	^B^
**MT10**	***trans*-roseoxide**	0.855 ± 0.296	^A^	-	^A^	1.51 ± 1.28	^A^	-	^A^	-	^A^	-	^A^	-	^A^
**MT11**	***trans*-linalooloxide (furanoid)**	23.9 ± 10.1	^A^	-	^B^	1.26 ± 0.791	^B^	0.174 ± 0.174	^B^	-	^B^	0.130 ± 0.130	^AB^	-	^AB^
**MT12**	***cis*-linalooloxide (furanoid)**	21.7 ± 6.97	^A^	-	^B^	1.43 ± 1.02	^B^	0.0783 ± 0.0783	^B^	-	^B^	0.106 ± 0.106	^B^	-	^AB^
**MT13**	**linalool**	461 ± 112	^A^	6.19 ± 4.97	^B^	3.45 ± 1.81	^B^	1.13 ± 0.515	^B^	-	^B^	2.90 ± 1.91	^B^	-	^B^
**MT14**	**hotrienol**	89.5 ± 27.5	^A^	2.52 ± 1.43	^B^	2.31 ± 1.69	^B^	-	^B^	-	^B^	-	^B^	-	^B^
**MT15**	**α-terpineol**	183 ± 65.8	^A^	3.11 ± 1.83	^B^	14.8 ± 8.55	^B^	8.73 ± 4.50	^B^	4.10 ± 1.28	^B^	1.34 ± 0.692	^B^	-	^AB^
**MT16**	**α-citral**	0.305 ± 0.249	^A^	-	^A^	-	^A^	-	^A^	-	^A^	-	^A^	-	^A^
**MT17**	***trans*-linalooloxide (pyranoid)**	134 ± 38.8	^A^	1.59 ± 1.45	^B^	1.3 ± 0.891	^B^	-	^B^	-	^B^	-	^B^	-	^B^
**MT18**	***cis*-linalooloxide (pyranoid)**	104 ± 49.2	^A^	0.122 ± 0.122	^B^	0.736 ± 0.507	^B^	-	^B^	-	^B^	-	^AB^	-	^AB^
**MT19**	**β-citronellol**	2.57 ± 2.31	^A^	-	^A^	-	^A^	-	^A^	-	^A^	-	^A^	-	^A^
**MT20**	**nerol**	11.3 ± 3.65	^A^	-	^B^	3.98 ± 3.21	^AB^	-	^B^	-	^B^	-	^AB^	-	^AB^
**MT21**	**guaniol**	34.6 ± 10.1	^A^	1.04 ± 0.716	^B^	1.96 ± 1.35	^B^	-	^B^	-	^B^	3.63 ± 3.63	^B^	6.64 ± 3.66	^AB^
**MT22**	**2,6-dimethyl-3,7-octadiene-2,6-diol**	1770 ± 596	^A^	29.6 ± 14.3	^B^	70.5 ± 28.4	^B^	8.77 ± 3.91	^B^	0.953 ± 0.756	^B^	0.617 ± 0.617	^B^	-	^AB^
**MT23**	**6,7-dihydro-7-hydroxylinalool**	43.4 ± 26.7	^A^	0.349 ± 0.349	^A^	2.27 ± 2.27	^A^	5.26 ± 5.26	^A^	-	^A^	0.977 ± 0.977	^A^	19.8 ± 4.54	^A^
**MT24**	**3,7-Dimethyl-1, 7-octadien-6-ol**	0.892 ± 0.479	^A^	-	^A^	-	^A^	-	^A^	-	^A^	-	^A^	-	^A^
**MT25**	**2,6-dimethyl-1,7-octadiene-3,6-diol**	35.2 ± 15.4	^A^	-	^B^	0.341 ± 0.341	^B^	-	^B^	-	^B^	-	^AB^	-	^AB^
**MT26**	**8-hydroxylinalool**	26.5 ± 10.6	^A^	-	^B^	-	^B^	-	^B^	-	^B^	-	^AB^	-	^AB^
**MT27**	**geranic acid**	147 ± 93.8	^A^	8.69 ± 4.89	^A^	7.60 ± 7.28	^A^	0.516 ± 0.311	^A^	-	^A^	-	^A^	31.0 ± 15.7	^A^
	**Monoterpenoids Subtotal**	3120 ± 883	^A^	53.8 ± 23.9	^B^	301 ± 89.3	^B^	239 ± 102	^B^	24.2 ± 8.84	^B^	17.3 ± 4.44	^B^	57.4 ± 18.3	^B^
	**Monoterpenoids Subtotal (%)**	58.3		6.25		4.81		9.12		0.684		0.103		0.0686	
**ST1**	**β-caryophyllene**	79.8 ± 69.5	^A^	2.92 ± 1.75	^A^	2.72 ± 2.2	^A^	0.254 ± 0.254	^A^	0.76 ± 0.621	^A^	-	^A^	-	^A^
**ST2**	**α-caryophyllene**	295 ± 225	^A^	4.18 ± 4.18	^A^	3.33 ± 2.83	^A^	-	^A^	-	^A^	-	^A^	-	^A^
**ST3**	**(*Z*)-β-farnesene**	636 ± 619	^A^	12.4 ± 7.89	^A^	3.23 ± 1.7	^A^	0.895 ± 0.613	^A^	-	^A^	-	^A^	-	^A^
**ST4**	**α-farnesene (1)**	0.203 ± 0.15	^A^	0.0878 ± 0.0878	^A^	721 ± 719	^A^	13.1 ± 7.80	^A^	-	^A^	10.4 ± 3.72	^A^	-	^A^
**ST5**	**α-farnesene (2)**	24.7 ± 21.2	^A^	0.987 ± 0.573	^A^	2940 ± 2760	^A^	68.1 ± 32.4	^A^	1.19 ± 1.11	^A^	59.9 ± 38.8	^A^	-	^A^
**ST6**	**calamenene**	71.4 ± 28.7	^A^	53.5 ± 14.1	^A^	214 ± 114	^A^	56.9 ± 27.1	^A^	30.3 ± 16.4	^A^	42.8 ± 16.1	^A^	95.6 ± 36.1	^A^
	**Sesquiterpenes Subtotal**	1110 ± 954	^A^	74.1 ± 21.8	^A^	3880 ± 3580	^A^	139 ± 53.0	^A^	32.2 ± 16.8	^A^	113 ± 40.1	^A^	95.6 ± 36.1	^A^
	**Sesquiterpenes Subtotal (%)**	20.7		8.61		62.2		5.31		0.911		0.671		0.114	
**N1**	**β-damascenone**	0.140 ± 0.140	^A^	4.71 ± 2.63	^A^	0.640 ± 0.640	^A^	1.44 ± 0.921	^A^	-	^A^	-	^A^	-	^A^
**N2**	**β-ionone**	5.80 ± 2.47	^B^	5.71 ± 1.47	^B^	2.65 ± 0.621	^B^	9.84 ± 4.04	^B^	2.24 ± 1.37	^B^	6.15 ± 2.64	^B^	34.2 ± 17.2	^A^
	**C**_**13**_ **Norisoprenoids Subtotal**	5.94 ± 2.46	^B^	10.4 ± 3.68	^B^	3.29 ± 0.912	^B^	11.3 ± 4.03	^B^	2.24 ± 1.37	^B^	6.15 ± 2.64	^B^	34.2 ± 17.2	^A^
	**C**_**13**_ **Norisoprenoids Subtotal (%)**	0.111		1.21		0.0527		0.431		0.0633		0.0365		0.0409	
	**The total Volatile contents**	5350 ± 1790	^B^	861 ± 131	^B^	6250 ± 3760	^B^	2620 ± 327	^B^	3530 ± 855	^B^	16 900 ± 4970	^B^	83 800 ± 21 800	^A^

All compounds were quantified as 3-heptanol equivalents. All values are quoted as mean ± standard error. ‘-’ indicates that the compound was not detected in the sample. Compounds that cannot be detected are considered to have a value of zero. Any two samples with a common uppercase letter within each flavour category are not significantly different (*p* > 0.05), according to the Tukey–Kramer HSD test.

### Volatile compounds associated with flavour characteristics through partial least squares analysis

Quantitative data from the analysis of 98 volatile compounds in 102 fresh grape samples were log-transformed and the relationship between volatile compound profiles and flavour characteristics was determined using the partial least squares (PLS) method. A model was developed for each flavour. Table 4 summarises the parameters of the PLS regression model. The cumulative R^2^Y and Q^2^ values of all flavour models except fresh green and floral exceeded the threshold of 0.5. Table 5 lists the 10 compounds with the highest VIP values for projection among foxy, muscat, and flavour intensity, as well as the compounds that were previously considered important for foxy and muscat flavours. In addition to volatile compounds with an OAV > 1, other compounds also exhibited high VIP values (Table 5).

**Table 4:** Partial least squares (PLS) regression model summary for each flavour

**Flavour category**	**Number of factors** ^**a**^	**Cumulative R** ^**2**^ **X** ^**b**^	**Cumulative R** ^**2**^ **Y** ^**b**^	**Cumulative Q** ^**2**^ ^**c**^	**Number of compounds (VIP > 1.0)** ^**d**^
**Intensity**	3	0.41	0.89	0.99	36
**Foxy**	2	0.30	0.89	0.97	40
**Muscat**	2	0.31	0.86	0.95	27
**Fruity**	1	0.21	0.61	0.57	36
**Sweet**	2	0.26	0.62	0.58	42
**Floral**	1	0.14	0.44	0.34	44
**Fermented/Sour**	2	0.28	0.78	0.89	39
**Fatty green**	1	0.21	0.62	0.57	46
**Fresh green**	1	0.21	0.29	0.21	39

All flavour categories were regressed on the log-transformed quantitative data of 98 volatile compounds in 102 samples of fresh grapes using PLS.

a Number of factors is the number that provides the best predictive performance on the validation set.

b Cumulative R^2^X and Cumulative R^2^Y report the percentage of X and Y variations explained by the model with the given number of factors.

c Cumulative Q^2^ is an indicator of the predictive ability of models with the given number of factors or fewer.

d Number of compounds (VIP > 1) indicates the number of compounds that are considered important for prediction. VIP = importance of the variable for projection.

**Table 5:** The 10 compounds with the highest VIP values in (A) muscat, (B) foxy, and (C) flavour intensity based on PLS analysis, and those compounds considered important for foxy and muscat flavour based on previous studies

**(A) Compounds related to muscat**	**Muscat**	**Intensity**	**Foxy**	**Fruity**	**Sweet**	**Floral**	**Fermented / Sour**	**Fatty Green**	**Fresh Green**
	**VIP & C**	**VIP & C**	**VIP & C**	**VIP & C**	**VIP & C**	**VIP & C**	**VIP & C**	**VIP & C**	**VIP & C**
*cis*-linalooloxide (pyranoid)	**+++**	**+**	**−**	**+**	**−**	**+**	**−**	**−**	**++**
8-hydroxylinalool	**+++**	**+**	**−**		**+**		**+**	**−**	**+**
*trans*-linalooloxide (pyranoid)	**+++**		**−**	**+**	**−**	**+**	**−**	**−**	**++**
2,6-dimethyl-1,7-octadiene-3,6-diol	**+++**		**−**		**+**		**+**	**−**	**+**
**linalool**	**+++**		**−**		**−**		**−**	**−**	**++**
hotrienol	**+++**		**−**		**+**	**+**	**−**	**−**	**+**
*trans*-linalooloxide (furanoid)	**+++**		**−**		**+**	**+**	**+**	**−**	**+**
*cis*-linalooloxide (furanoid)	**+++**		**−**		**+**	**+**		**−**	**++**
***cis*-rose oxide**	**++**		**−**		**+**	**+**	**+**	**−**	**+**
2,6-dimethyl-3,7-octadiene-2,6-diol	**++**		**−**	**+**	**+**		**+**	**−**	**+**
nerol	**++**					**+**			**+**
guaniol	**++**					**+**			**+**
α-terpineol	**+**				**+**	**+**			**+**
***trans*-roseoxide**	**+**					**+**			
**(B) Compounds related to foxy**	**Foxy**	**Intensity**	**Muscat**	**Fruity**	**Sweet**	**Floral**	**Fermented / Sour**	**Fatty Green**	**Fresh Green**
	**VIP & C**	**VIP & C**	**VIP & C**	**VIP & C**	**VIP & C**	**VIP & C**	**VIP & C**	**VIP & C**	**VIP & C**
ethyl 3-hydroxyhexanoate	**++**	**++**	**−**	**++**	**+**	**+**	**++**	**+**	**−**
ethyl octanoate	**++**	**++**	**−**	**++**	**+**	**+**	**++**	**++**	**−**
**ethyl decanoate**	**++**	**++**	**−**	**++**	**+**	**++**	**++**	**++**	**−**
ethyl 3-hydroxybutanoate	**++**	**++**	**−**	**++**	**+**	**++**	**++**	**++**	**−**
**ethyl hexanoate**	**++**	**+**	**−**	**++**	**+**	**++**	**+**	**+**	**−**
**ethyl (*E,Z*)-2,4-decadienoate**	**++**		**−**	**+**	**+**	**+**	**+**	**+**	
ethyl 2-butenoate	**++**	**+**	**−**	**++**	**+**	**++**	**+**	**+**	**−**
**ethyl heptanoate**	**++**	**+**		**+**	**−**		**+**	**+**	**−**
ethyl benzeneacetate	**++**						**+**	**+**	
ethyl *trans*-4-decenoate	**++**	+	−	+	−	+	++	+	++
**mesifurane**	**+**	**++**		**+**			**+**	**++**	**−**
methyl *N*-formylanthranilate	**+**	**+**			**−**			**+**	
**furaneol**		**+**		**+**	**+**		**+**	**++**	
methyl anthranilate									
**(C) Compounds related to flavour intensity**	**Intensity**	**Foxy**	**Muscat**	**Fruity**	**Sweet**	**Floral**	**Fermented / Sour**	**Fatty Green**	**Fresh Green**
	**VIP & C**	**VIP & C**	**VIP & C**	**VIP & C**	**VIP & C**	**VIP & C**	**VIP & C**	**VIP & C**	**VIP & C**
**β-phenethyl acetate**	**++**	**+**		**++**	**−**	**+**	**+**	**++**	**−**
ethyl 3-hydroxybutanoate	**++**	**++**	**−**	**++**	**+**	**++**	**++**	**++**	**−**
ethyl octanoate	**++**	**++**	**−**	**++**	**+**	**+**	**++**	**++**	**−**
methyl 3-hydroxybutanoate	**++**	**+**		**++**	**+**	**+**	**+**	**++**	
**mesifurane**	**++**	**+**		**+**			**+**	**++**	**−**
**ethyl decanoate**	**++**	**++**	**−**	**++**	**+**	**++**	**++**	**++**	**−**
**ethyl cinnamate**	**++**			**+**	**−**		**+**	**+**	
ethyl 3-hydroxyhexanoate	**++**	**++**	**−**	**++**	**+**	**+**	**++**	**+**	**−**
**furaneol**	**+**			**+**	**+**		**+**	**++**	
butyl octanoate	**+**			**+**		**+**		**+**	**+**

C: coefficient, +: VIP > 1 and positive contribution, ++: VIP > 1.5 and positive contribution, +++: VIP > 2.0 and positive contribution, −: VIP > 1 and negative contribution, —: VIP > 1.5 and negative contribution, —: VIP > 2.0 and negative contribution

Positive and negative contributions are shaded in pink and blue, respectively, with the intensity of the colour indicating the value of VIP, and VIP < 1.0 is indicated by a white background.

Bold text in (A) indicates the active volatile compounds (OAVs >1) identified in cluster 1. Bold text in (B) indicates the active volatile compounds (OAVs >1) identified in clusters 5 or 6. Bold text in (C) indicates the active volatile compounds (OAVs >1) identified in any of clusters 1–7. The respective active volatile compounds were obtained from Supplemental Table S5.

This study identified 27 compounds as key variables in the muscat flavour with VIP values greater than 1 (Table 4). Eighteen monoterpenoids (including linalool, geraniol, nerol, α-terpineol, and *cis*-rose oxide) and α-caryophyllene were positively associated with the muscat flavour (Table S3). Linalool derivatives (including linalool oxide and 8-hydroxylinalool) showed the strongest association with the muscat flavour (Table 5A), while nine ethyl esters (including ethyl octanoate, ethyl decanoate, ethyl-3-hydroxybutanoate, ethyl-2-butenoate, ethyl hexanoate, and ethyl butanoate) exhibited a negative association with the muscat flavour (Table S3). Forty compounds were identified as key variables in the foxy flavour with VIP values greater than 1 (Table 4), whereas the VIP values of furaneol and methyl anthranilate did not exceed 1 (Table 5B). Seventeen ethyl esters (including ethyl-3-hydroxyhexanoate, ethyl octanoate, ethyl decanoate, ethyl-3-hydroxybutanoate, ethyl hexanoate, and ethyl (*E,Z*)-2,4-decadienoate) and 11 other compounds (including methyl-3-hydroxybutanoate, β-phenethyl acetate, acetoin, methyl salicylate, and β-pinene) were positively associated with the foxy flavour (Table S3). Methyl *N*-formylanthranilate, which has a green floral-like aroma similar to that of Concord grapes, and mesifurane, described as having a burned, sherry-like, or fusty aroma, were also associated with the foxy flavour (Table 5B). Eleven monoterpenoids (including *trans*-linalooloxide (pyranoid), hotrienol, linalool, and *cis*-rose oxide, *etc*.) and 3-hexenal showed a negative associated with the foxy flavour (Table S3). Compounds related to muscat and foxy flavours also influenced the flavour intensity and several other flavour characteristics (Table 5A and 5B). Compounds that were strongly associated with the muscat flavour (linalool, linalool derivatives, and monoterpenoids) were positively related to floral and fresh green flavours, and negatively relate to foxy and fatty green flavours (Table 5A). Compounds that were strongly associated with the foxy flavour (ethyl esters) were positively related to flavour intensity and the fruity, sweet, floral, fermented/sour, and fatty green flavours, but were negatively associated with muscat and fresh green flavours (Table 5B). Compounds found only in muscadines, such as butyl, hexyl, and phenethyl esters, were related to flavour intensity, positively associated with the fruity, sweet, floral, fermented/sour, and fatty green flavours, and negatively associated with the fresh green flavour (Table 5C, Table S3). Methyl-3-hydroxybutanoate, ethyl cinnamate, phenylethyl alcohol, mesifurane, furaneol, α-pinene, and β-pinene were not strongly associated with the foxy and muscat flavours; however, these compounds were strongly associated with the flavour intensity and fruity, fatty green, sweet, floral, and fermented/sour flavours (Table 5C, Table S3).

## Discussion

A large-scale sensory and chemical analysis of 102 grape samples was conducted over three years to identify the volatile compounds responsible for grape flavour. Our study analysed grape flavour by sensory evaluation and volatile compound profiles obtained using the SAFE method. Furthermore, this study builds upon previous research [9] by using a large number of samples incorporating a greater variety of flavours, and provides new insights into the volatile compounds that contribute to grape flavour.

### Profiles of a wide variety of flavour characteristics in table grapes

Flavour characteristics are typically determined by sensory evaluation. The flavour characteristics of ripe table grapes include muscat, foxy, muscadine, and neutral [5, 7, 14]. In this study, a flavour wheel including foxy, muscat, fresh green, fatty green, fermented/sour, floral, sweet, and fruity flavours, among others, was created for table grapes (Fig. S1). This flavour wheel includes categories similar to those used by Sasaki *et al.* in the sensory evaluation of table grapes [22], along with additional categories such as fresh green, fatty green, fermented, and muscat flavours. Our findings demonstrate that the developed flavour wheel facilitates the classification and evaluation of the flavour characteristics of table grapes and will therefore expedite the future analysis of grape flavour profiles.

Cluster analysis of the sensory evaluation data of the conventional elements of flavour classification, *i.e.,* foxy flavour, muscat flavour, and flavour intensity, produced seven clusters (Fig. 1). The classification of the conventional flavour characteristics of muscat, none, and muscadine formed clusters; however, the remaining flavours, including foxy, formed multiple clusters, indicating their diversity (Fig. 1). A possible factor in the formation of multiple clusters was the diversity of foxy flavour and flavour intensity among the samples is a contributing factor in the formation of multiple clusters. In particular, certain samples in cluster 3 exhibited sweet, floral, and fruity flavours rather than muscat and foxy flavours (Fig. 1, Table S2). Interestingly, grapes of the same cultivar grown in different years tended to cluster together, highlighting the significant influence of genetic factors on flavour characteristics (Fig. 1).

### Analysis of volatile compounds using SAFE

The application of SAFE in the analysis of the volatile compounds of grapes is limited. To the best of our knowledge, the present study represents the first use of SAFE to obtain volatile profiles in table grapes, despite its use for this purpose in the analysis of wine [29]. We detected C_6_ compounds (including hexanal, (*E*)-2-hexenal, and 3-hexenol), abundant esters (such as ethyl butanoate and ethyl hexanoate), and monoterpenoids (such as linalool, geraniol, nerol, citronellol, and α-terpineol) in table grapes (Table 2). While earlier volatile profiling studies of table grapes also identified these compounds [16, 18], we also observed intermediate-molecular-weight esters and acids, hydroxyl esters, hydroxyl monoterpenoids and polyols, furaneol, methyl anthranilate, methyl *N*-formylanthranilate, acetoin, and vanillin (Table 2), which have rarely been detected by the HS-SPME analysis of the volatile compounds in table grapes [16, 18]. Further, to the best of our knowledge, no other study has detected methyl *N*-formylanthranilate in grapes, which may be produced by enzymatic or chemical reactions involving the formylation/deformylation of methyl anthranilate and its analogues in the fruit. These observations suggest that the SAFE method provides a comprehensive analysis of the volatile compounds, including trace compounds and those with high boiling points, in table grapes and in other horticultural crops [25, 26, 29].

### Relationship between diversities of volatile compounds and flavour characteristics of table grapes

Several esters (particularly ethyl esters) and monoterpenoids contributed to the differences in main volatile compounds found in *V. vinifera*, *V. rotundifolia*, and *V. interspecific crossing* (Fig. 2A and 2D). These results were partially consistent with those of a previous study [16], which found that terpenoids are prevalent in *V. vinifera* cultivars with muscat aroma, while esters are the most abundant in *V. labrusca* and its hybrids with *V. vinifera* or *V. amurensis*. Furthermore, both *V. rotundifolia* and *V. interspecific* crosses exhibited varied volatile profiles that include more than ethyl esters and monoterpenoids (Fig. 2). *V. rotundifolia* contained hexyl, butyl, and phenylethyl esters and the corresponding alcohols (Fig. 2A and 2D), while *V. interspecific crossing* exhibited a wide variety of volatile profiles: one volatile profile was similar to that of *V. vinifera* varieties with muscat flavour, such as ‘Shine Muscat’ (Fig. 2A and 2D, Fig 3). Certain varieties such as Keuka had a mixed volatile profile of ethyl esters and monoterpenoids (Fig. 2B and 2E, Fig 3). In contrast, other varieties such as ‘Muscat Berry A’ and ‘Delaware’, which are *V. interspecific crossing* with the genetic backgrounds of *V. lincecomii* and *V. amurensis*, respectively, had lower levels of esters and monoterpenoids but higher levels of farnesene, furaneol, and mesifurane (Fig. 2C and 2F, Fig 3). These results suggest that the diversity of volatile components in table grapes arises from differences in the content and composition of not only ethyl esters and monoterpenoids, but also those of other compounds like hexyl, butyl, phenylethyl esters, their corresponding alcohols, and furanones, such as furaneol and mesifurane. The diversity of volatile profiles in table grapes is likely related to the genetic background. Interestingly, the first four principal components (PC1, PC2, PC3, and PC4) were consistent with the flavour classification obtained by sensory evaluation (Fig. 2), suggesting that the diversities in flavour characteristics and volatile compounds are interrelated.

### Search for volatile compounds associated with flavour characteristics using PLS analysis

The relationship between sensory descriptors and volatile compounds was determined using SAFE-GC–MS and PLS. For the *Q*^2^ parameter, a significance threshold of 0.5 generally indicates that the model has a high explanatory power [30]. All flavour models developed here, except for those of fresh green and floral, exhibited high explanatory power (Table 4). The models of foxy flavour, muscat flavour, and flavour intensity were particularly effective; thus, volatile compounds analysed by SAFE-GC/MS are expected to accurately predict the grape flavour, particularly in the case of grapes with a foxy or muscat flavour. PLS-VIP identified the volatile compounds in grapes that are responsible for flavour characteristics. A parameter with a higher VIP value is more important to the model; parameters with VIP values greater than 1 are important compound flavour characteristic [9, 24, 31]. Analysis of each flavour characteristic identified between 27 and 46 compounds with VIP values greater than 1, many of which showed association with flavour characteristics (Table 4). This indicates that multiple compounds are involved in the formation of flavours. In addition, compounds can be associated with different flavours; for example, the ethyl esters that contribute to the foxy flavour and monoterpenoids that contribute to the muscat flavour were overlappingly associated with other flavours, including fruity and floral (Table 5, Table S3). This suggests that flavours, such as foxy and muscat, are supported by the complex combination of flavours. We therefore summarize the volatile compound profiles of representative flavour categories based on sensory evaluation.

### Characteristics of flavour and volatile compounds in cluster 1, which primarily contains Muscat varieties

‘Muscat’ is known for its distinct floral flavour [19]. Cluster 1, which primarily includes muscat varieties, had muscat, floral, fresh green, and sweet flavours with flavour intensities above three (Table S2). The most abundant monoterpenoids linalool, nerol, and geraniol are the primary flavour compounds responsible for the muscat flavour [32, 33]. Monoterpenoids were present at approximately 3.12 mg·kg^−1^ FW and account for 58.3% of the composition in cluster 1 (Table 3). Twenty-seven compounds including linalool, nerol, and geraniol exhibited a positive association with the muscat flavour (Table S3). In particular, linalool and its derivatives (*cis*-linalooloxide (pyranoid), 8-hydroxylinalool, *trans*-linalooloxide (pyranoid), and 2,6-dimethyl-1,7-octadiene-3,6-diol) exhibited the strongest association (Table 5). Interestingly, linalool and its derivatives showed a positive relationship with the fresh green flavour, while linalool derivatives, α-terpineol, and *cis*/*trans*-roseoxide exhibited positive relationship with the floral flavour (Table 5). Cluster 1 showed significantly higher linalool, hotrienol, *trans*-linalooloxide (pyranoid), and *cis*-rose oxide contents than the other flavour characteristic clusters (Table 3). These compounds can therefore be considered as markers of muscat, fresh green, and floral flavours in table grapes. Ruiz-García *et al.* [34] also suggested that the correlation between rose oxide and muscat flavour could be a useful tool in the identification and selection of table grapes. Furthermore, linalool and *cis*-rose oxide have OAV values greater than 1 (Table 5, Table S5), suggesting their potential influence on the perception of muscat, fresh green, and floral flavours in muscat varieties.

### Characteristics of flavour and volatile compounds in clusters 5 and 6, which primarily contain foxy varieties

The term ‘foxy’ describes the unique, earthy, and sweet muskiness present in most *V. labrusca* and *V. rotundifolia* species [35]. Clusters 5 and 6 primarily contained foxy varieties and had high levels of foxy, fruity, fermented/sour, fatty green, floral, and sweet flavours with flavour intensities above three (Table S2).

At least three compounds contribute to the foxy flavour: methyl anthranilate, 2-aminoacetophenone, and furaneol [20, 21]. In this study, methyl anthranilate was detected at a frequency of 6.25% in cluster 5 and 12.5% in cluster 6, while furaneol was detected at 100% in cluster 6 but was absent in cluster 5 (Table S4). Baek *et al*. detected 2-aminoacetophenone with in ‘Carlos’ samples with the foxy flavour [21], but this compound was not identified in the current study. Interestingly, 28 compounds, including 17 ethyl esters (Table S3), were more positively associated with the foxy flavour than either methyl anthranilate and furaneol, neither of which showed associations with the foxy flavour (Table 5). Some of these compounds, including ethyl 3-hydroxyhexanoate, ethyl octanoate, ethyl hexanoate, ethyl butanoate, and ethyl 3-hydroxybutanoate, are known to influence the fruity aroma of pears, strawberries, pineapple, and wine [36, 37]. Because those compounds were detected in most samples of clusters 5 and 6, they are likely important contributing factors to the foxy flavour of hybrid grapes.

Despite both being considered foxy varieties, clusters 5 and 6 exhibit some differences (Fig. 1). Cluster 5 includes varieties described as favourable foxy, while samples in cluster 6 have significantly higher values for foxy and fatty green flavours (Table S2). The contents of 18 compounds, including methyl *N*-formylanthranilate, mesifurane, methyl 3-hydroxybutanoate, acetic acid, and several ethyl esters in clusters 5 and 6 differed significantly (Table 3). All compounds except *β*-phellandrene were more abundant in cluster 6 than cluster 5. Like methyl anthranilate and furaneol, methyl *N*-formylanthranilate and mesifurane were detected at low frequencies (6.25%) in cluster 5 but high frequencies (75%–100%) in cluster 6 (Table S4). Further, methyl *N*-formylanthranilate has green floral aromas reminiscent of ‘Concord’ grapes, while mesifurane has aromas reminiscent of strawberries, burned sugar, and caramel. The relative abundance of these 18 compounds may contribute to the flavour diversity and preference of the foxy varieties.

### Characteristics of flavour and volatile compounds in cluster 7, which primarily contains muscadine varieties

Muscadine grapes are known for their dense, foxy, candy like, and uniquely fruity aromas [21, 24]. Cluster 7, which includes most muscadine varieties, showed foxy, fruity, fermented/sour, fatty green, floral, and sweet flavours, which were more intense and richer than those of the other clusters (Table S2). The total compound content of cluster 6 was approximately 5–100 times higher than that of other clusters (Table 3). Some compounds identified by Baek *et al.* [21] as the most abundant aroma compounds in muscadine juice (furaneol, ethyl butanoate, and phenylethyl alcohol) accumulated and thus increased the flavour intensity (Table 5, Table S3); however, the most abundant compounds were alcohols (35.2 mg·kg^−1^ FW, 42.0%) and esters other than ethyl esters (45.1 mg·kg^−1^ FW, 53.8%) (Table 3). Ten compounds (*i.e.,* 1-hexanol, octanol, phenylethyl alcohol, hexyl hexanoate, β-phenethyl acetate, hexyl octanoate, phenethyl hexanoate, phenylethyl octanoate, benzyl acetate, and phenylacetaldehyde) were significantly more abundant in cluster 7 than in the other clusters and associated with flavour intensity (Table 3, Table S3). In addition, isoamyl alcohol, benzyl alcohol, γ-hexalactone, and β-ionone were significantly more abundant in cluster 7 than in the other clusters, although they were not strongly associated with flavour intensity (Table 3, Table S3). These compounds have green, fruity, floral, and waxy aromas, which may contribute to the unique flavour of muscadine.

### Characteristics of flavour and volatile compounds in clusters 2, 3, and 4, which primarily contain neutral varieties

Cluster 2 primarily contains cultivars classified as ‘neutral’ flavour, and has the lowest abundance and number of detected compounds; 46 compounds with a total content of 860 mg·kg^−1^ FW were detected (Table 3). The C_6_ compounds, aldehydes, ketones, and alcohols accounted for 72.8% of the volatile compounds present in cluster 2 (Table 3). C_6_ compounds (hexanal, (*E*)-2-hexenal, and 3-hexenol), also known as ‘green leaf volatiles’, are basic background volatiles in table grape berries [16, 23]. Like the C_6_ compounds, acetic, hexanoic, nonanoic, and benzoic acids, along with benzyl alcohol, benzaldehyde, and vanillin are considered basic background volatiles because they were detected in 90% of all samples (Table 2). The contents of volatile compounds, including the basic background volatiles, differ significantly among table grape samples [23]; however, we did not observe any significant differences in the content of these compounds among the clusters (Table 3). These basic background compounds were present in cluster 2 and might contribute to the grape flavour; however, the lack of any distinctive relationship between the compounds present and the flavours may have resulted in a less flavourful cluster relative to the other clusters.

Clusters 3 and 4, consisting mostly of specific-flavoured varieties, were characterized by flavours other than foxy and muscat, including sweet, fruity, floral, fatty green, and fermented/sour flavours (Fig. 1, Table S2). Clusters 3 and 4 exhibited more diverse volatile compound profiles than the other clusters owing to the higher number of compounds in these two clusters (75 and 71 compounds, respectively). Additionally, clusters 3 and 4 contained a higher number of compounds with a frequency ratio of less than 50% (48 and 37 compounds, respectively) than the other clusters (5–27 compounds, Table S4). Some specific cultivars, such as ‘Keuka’, ‘Buffalo’, ‘Delaware’, and ‘Muscat Bailey A’, exhibited distinct compound profiles. For example, ‘Keuka’ contained a mixture of ethyl esters and monoterpenoids, while ‘Buffalo’ and ‘Delaware’ specifically accumulated farnesene, furaneol, mesifurane, and γ-decalactone (Fig. 3). Like ‘Keuka’, the accumulation of both monoterpenoids and ester compounds produced a distinct flavour without intensifying the muscat or foxy flavour owing to the negative relationships between muscat flavour and esters and between foxy flavour and monoterpenoids (Table 5). Along with mesifuran, furaneol, which is responsible for the strawberry-like aroma of ‘Muscat Bailey A’, contributes to the typical caramel-like, sweet, floral, and fruity aroma [36, 38]. Additionally, fatty green, fruity, and sweet flavours were associated with the unique flavour profiles in clusters 3 and 4 (Table 5, Table S3). Although farnesene and γ-decalactone were less strongly associated with the flavour intensity (Table S3), the sweet or peachy flavour of γ-decalactone and the woody odour of *α*-farnesene have been proposed as descriptors for classifying apple cultivars [36]. Thus, sensory evaluations of grape flavours in clusters 3 and 4 correspond to specific compound profiles, suggesting that these cultivars can be used to efficiently breed novel flavours and preferred cultivars owing to the diversity of their compound profiles and flavours.

### Interaction of aroma compounds

Fruits contain numerous volatile compounds that contribute to their diverse sensory perceptions. The qualitative (odour quality) and quantitative (odour intensity) sensory perceptions of these compounds interact in various ways to create the overall sensory experience of the fruit [39–42].

The OAV, defined as the concentration-odour threshold ratio, is commonly used to assess the contribution of each compound to fruit aroma. Compounds with OAVs greater than one are considered active contributors [9, 19, 23]. Compounds identified as active contributors, including linalool, ethyl hexanoate, ethyl (*E,Z*)-2,4-decadienoate, *β*-phenethyl acetate, phenethyl alcohol, furaneol, mesifurane, and other compounds, were also associated with flavour (Table 5, Table S5). Some linalool derivatives, hydroxyl esters, and other compounds exhibited even stronger associations (Table 5), but the OAVs of these compounds are either below the threshold or without available threshold information. Despite showing OAVs below the threshold, linalool oxides enhance the perception of muscat aroma, and ethyl 3-hydroxybutanoate enhances that of fruity flavour in wine and juice [32, 37, 41]. This demonstrates that approaches based solely on odour thresholds are insufficient, because compounds at or below the threshold may still affect the flavour of table grapes. The role of volatiles in grape flavour therefore depends on both their OAVs and their interactions with other compounds. Furthermore, to identify compounds that contribute to grape flavour, the complex interactions of flavour-related compounds must be evaluated, including taste, texture, and threshold information.

## Conclusion

This study provided detailed flavour characteristics and volatile compound profiles of the main grape varieties grown as table grapes and genetic resources. Sensory evaluation using a flavour wheel including flavours other than foxy and muscat revealed that table grapes have a variety of flavour characteristics such as sweet, floral, and fruity. SAFE provided comprehensive profiles of volatile compounds in grapes, including those of slightly volatile substances that have not been reported before. Multivariate analysis using flavour characterization and volatile compound data identified compounds strongly correlated with flavour in addition to those identified based on the concentration of individual volatiles and odour thresholds. Both volatile compound analysis with SAFE and multivariate analysis not based on conventional odour units can identify novel classes of flavour compounds in table grapes. This fundamental knowledge is expected to enable more efficient creation of novel grape cultivars with highly preferred flavours and more accurate methods to assess the flavour quality.

## Materials and methods

### Grape samples

Grapes (*Vitis* spp.) were grown in vineyards at the Grape and Persimmon Research Station (NARO, Japan). The vine ages of the evaluated cultivars were between three and 30 years as of 2017. Vine management such as fertilization and canopy management were performed according to the usual practices in Japan, and the vines were cane-pruned. Kober (Teleki) 5BB was used as the rootstock. A total of 102 fruit samples were harvested from 38 table grape cultivars in 2017, 2018, and 2019 when the acid content was judged to have decreased enough by sensory evaluation (Table S1). Bunches of grapes from each sample were prepared for sensory evaluation and chemical analysis. The harvested grapes were transported to the laboratory at the Grape and Persimmon Research Station for sensory evaluation and sugar and acidity analysis. For volatile compound analysis, the fruits were immediately transported under refrigeration to the University of Tsukuba and stored at 5°C. The storage period from harvest to extraction was approximately three days.

### Sensory analysis by expert panels

Eleven individuals experienced in flavour development (six males and five females) provided a list of 203 terms to describe the flavour of grapes (‘Pione’, ‘Kyoho’, ‘Delaware’, ‘Shine Muscat’, and ‘Seto Giants’). A flavour wheel was created by defining and sharing evaluation terms as classifications using the KJ method with six individuals experienced in flavour development and four sensory evaluators (seven males and three females) (Fig. S1). Over the course of 3 years, evaluation was conducted by four grape breeders individually each year. In the first 2 years (2017 and 2018), evaluation was conducted by the same four panels. In the third year (2019), one panel was replaced and the other three panels were unchanged. Evaluation was performed at the appropriate harvest time for each variety, and no specific time interval was set between evaluations of different varieties. The grapes of each variety were prepared in a tray in the research room, and each panel performed sensory evaluation using multiple cultivars and multiple grape berries. Each panel evaluated each flavour descriptor (flavour intensity, foxy, muscat, fresh green, fatty green, fermented/sour, floral, sweet, and fruity) on a seven-point scale (extremely weak, very weak, weak, moderate, strong, very strong, and extremely strong) using the format shown in Fig. S2, on the degree of each flavour by reference to the evaluation term shown in Fig. S1. Examples of the flavour intensity of certain varieties were provided in Fig. S2 as a guide. For example, the flavour intensity of ‘Kyoho’ (the leading Japanese variety) is set as four (Moderate).

### Extraction method

Volatile compounds were analysed using a partial modification of the SAFE method of Engel et al [25] as follows. Three bunches of grapes from each sample were washed with distilled water. After drying the surface, at least 18 berries, selected equally from the three bunches, were sliced into quarters, de-seeded and randomly selected to obtain 100 or 300 g of fruit. Before grinding the fruit in a juicer, saturated calcium chloride solution was added at a ratio of approximately 0.8 times the weight of the fruit, along with 0.5 or 0.15 ml of an internal standard (3-heptanol at 0.1 mg·ml^−1^). Dichloromethane was added to the juice at approximately 0.8 times the weight of the fruit, and the volatile compounds were extracted by stirring for 1 h. The extract was isolated using SAFE (Kiriyama Glass Works Co.) at 35°C under vacuum (3 × 10^−2^ Pa), and subsequently concentrated under vacuum at 35°C using a high-vacuum pumping system (VPC-250F, ULVAC KIKO, Inc.). Finally, the extracts were dried over sodium sulfate and concentrated to 0.5 ml using a rotary evaporator.

### GC–MS analysis of volatile compounds

GC–MS analysis was performed using a Focus GC/DSQII (Thermo Fisher Scientific) equipped with DB-WAX UI capillary column (0.25 mm × 0.25 μm × 30 m, Agilent). The concentrated extract obtained using the SAFE method (2 μl) was injected in split mode (1:10) under a constant helium flow of 1.0 ml·min^−1^. The injector was maintained at 250°C. The column oven temperature was initially maintained for 5 min at 40°C for 5 min, then further heated to 160°C at a rate of 2°C·min^−1^ to 160°C, and finally to 240°C at a rate of 4°C·min^−1^ and maintained for 10 min. Mass spectra were obtained by electron impact ionization (70 eV) by scanning in the mass range of *m/z* 35–600. The MS transfer line and ion source temperatures were 250°C. The retention indices were calculated by analysing C_7_–C_32_ n-alkanes under the same chromatographic conditions for identification. The volatile compounds were tentatively identified using a chemical standard, retention index, or library (NIST MS Search 2.0). When authenticated standards were not available, tentative identification was based on the library (NIST MS Search 2.0) or NIST Chemistry WebBook [43] and a comparison to retention indices reported in the literature. All compounds were quantified as 3-heptanol equivalents. The OAV was calculated using concentration (equivalent of 3-heptanol) /threshold, and odour thresholds for the volatile compounds were obtained from previous reports (Table S5).

### Data analysis

Sensory evaluation data was reported as the mean score from four panels. Volatile compounds data were obtained in a single measurement without repetition. All statistical processing was performed using JMP14 software (SAS Institute Inc., Cary, NC, USA). Significant variances were validated using one-way ANOVA and Tukey’s HSD. PCA was performed by row-wise estimation using log-transformed volatile compound data. HCA was performed by Ward’s method using sensory evaluation. Each flavour (Y-variable) was regressed on the log-transformed volatile compounds data (X-variables) using the PLS. PLS was performed using the NIPALS method and leave-one-out cross-validation to extract the minimum number of factors with van der Voet significance greater than 0.10 [31, 44, 45]. Centring and Scaled coefficients and VIPs for that number of factors were determined.

## Acknowledgements

This research was supported by grants from the Project of the Bio-oriented Technology Research Advancement Institution, NARO (special scheme project on advanced research and development for next-generation technology) and the project ‘Support for Pioneering Research Initiated by the Next Generation (SPRING)’ (grant no. JPMJSP2124) commissioned by Japan Science and Technology Agency. The authors acknowledge Ms. Miho Kohata for technical assistance. The authors are grateful to Dr. Shigeru Matsuyama for providing chemical samples and analytical advice. They also thank Mr. Masaya Kono, Mr. Yuuhi Hattori, Ms. Yuriko Imayoshi, and Dr. Hisakatsu Iwabuchi (Saneigen FFI) for their technical assistance and advice on flavour extraction using the SAFE method and sensory evaluation.

## Author Contributions

K.M. and S.S. designed the study. K.M., A.K., N.O., R.M., A.A., S.A., and S.S. contributed to the discussion on experimental design, grape sampling for chemical analysis and for sensory evaluation. K.M. performed chemical analyses and data analyses. K.M., A.K., N.O., and S.S. made significant contributions to the design of the sensory evaluation. A.K. and N.O. organized and directed the sensory panel. A.K., N.O., R.M., A.A., and S.A. helped collect the sensory evaluation data. K.M., A.K., N.O., Y.S., S.A., and S.S. contributed to the discussion on data interpretation. S.S. oversaw the whole project. K.M. wrote the manuscript and S.S. edited it. All authors critically reviewed the draft manuscript on the intellectual content. All authors read and approved the final manuscript.

## Data availability

The data supporting the findings of this study are included in this article and supplementary material.

## Conflict of interests

The authors declare that they have no known competing financial interests or personal relationships that could have appeared to influence the work reported in this paper.

## Supplementary information

Supplementary data is available at Horticulture Research online.

## Supplementary Material

Web_Material_uhae048
