# Supplementary material for: Diversity of flavour characteristics of table grapes and their contributing volatile compounds analysed by the solvent-assisted flavour evaporation method
Source: Hortic Res. 2024 Feb 26;11(4):uhae048. doi: 10.1093/hr/uhae048 (PMC11031413; doi:10.1093/hr/uhae048)
Supplement: Web_Material_uhae048 [file web_material_uhae048.zip › Fig. S1..pdf]

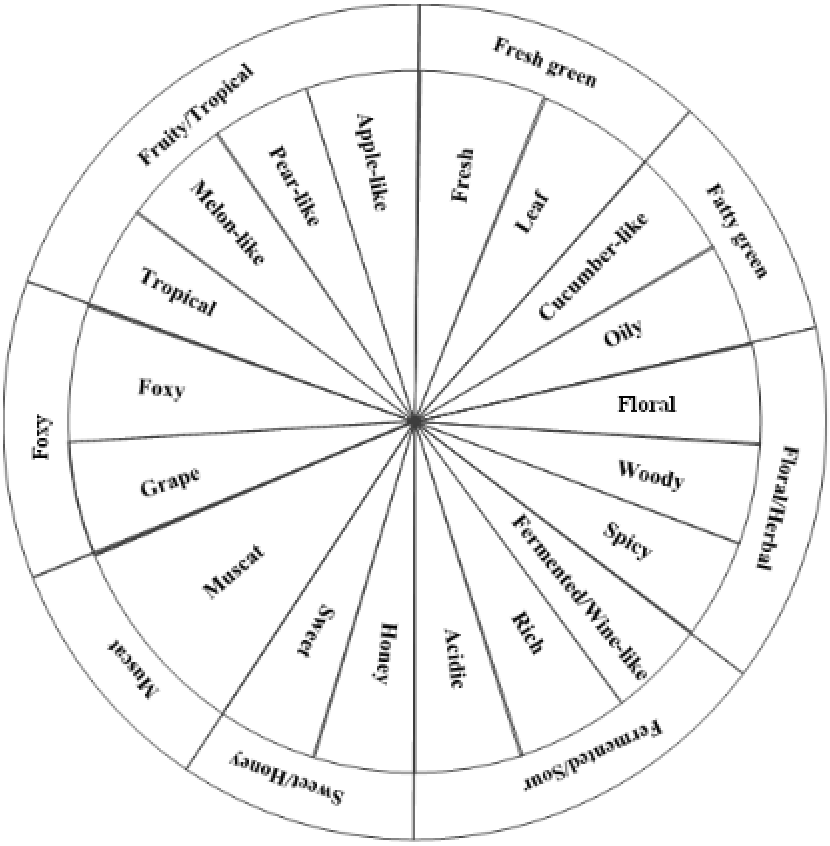

Flavour descriptor	Evaluation terms
Fresh green	fresh, leafy, green, vegetable, green grass, wasabi, beans
Fatty green	waxy, oily green, stink bug-like scent, half-dried clothes
Floral/Herbal	Black tea, citrus, rose tone, gorgeous, jasmine, tree, tree branch-like, astringent, spicy, phenolic, like Seirogan (Japanese medicine brand name), medicine, rose, ramune sweets, lemon, jasmine, herbs, mint
Fermented/Sour	light acid, sharp, refreshing, heavy acid, (yogurt-like)sake aroma, alcohol, pickle-like, aged smell, mellow, full-bodied, heavy, strawberry, berry
Sweet/Honey	vanilla, caramel-like, sugar incense, sugar, sticky sweet, phenethyl-like, honey-like sweetness, sweet scent, heated candy, cotton candy
Foxy	total aroma of grapes (American grapes), grape odour
Muscat	total aroma of muscat
Fruity/Tropical	apple, aesthetic, green fruity, glue, tropical fruit, pineapple, strawberry, berry
