# Supplementary material for: Diversity of flavour characteristics of table grapes and their contributing volatile compounds analysed by the solvent-assisted flavour evaporation method
Source: Hortic Res. 2024 Feb 26;11(4):uhae048. doi: 10.1093/hr/uhae048 (PMC11031413; doi:10.1093/hr/uhae048)
Supplement: Web_Material_uhae048 [file web_material_uhae048.zip › Fig. S2.pdf]

Flavour descriptor	Extremely weak<br>1	Very weak<br>2	Weak<br>3	Moderate<br>4	Strong<br>5	Very strong<br>6	Extremely strong<br>7
**Fresh green**							
**Fatty green**							
**Floral/Herbal**							
**Fermented/Sour**							
**Sweet/Honey**							
**Foxy**							
**Muscat**							
**Fruity/Tropical**							

	Extremely weak<br>1	Very weak<br>2	weak<br>3	Moderate<br>4	strong<br>5	Very strong<br>6	Extremely strong<br>7
**Flovour intensity**							
Example varieties		Sun Verde		Kyoho		Campbell Early	
