# Supplementary material for: Diversity of flavour characteristics of table grapes and their contributing volatile compounds analysed by the solvent-assisted flavour evaporation method
Source: Hortic Res. 2024 Feb 26;11(4):uhae048. doi: 10.1093/hr/uhae048 (PMC11031413; doi:10.1093/hr/uhae048)
Supplement: Web_Material_uhae048 [file web_material_uhae048.zip › Supplemental data.docx]

**Supplemental data**


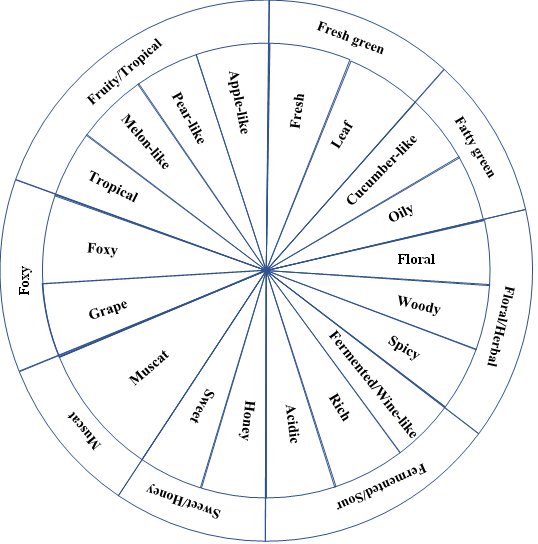


| **Flavour descriptor** | **Evaluation terms** |
| --- | --- |
| Fresh green | fresh, leafy, green, vegetable, green grass, wasabi, beans |
| Fatty green | waxy, oily green, stink bug-like scent, half-dried clothes |
| Floral/Herbal | Black tea, citrus, rose tone, gorgeous, jasmine, tree, tree branch-like, astringent, spicy, phenolic, like Seirogan (Japanese medicine brand name), medicine, rose, ramune sweets, lemon, jasmine, herbs, mint |
| Fermented/Sour | light acid, sharp, refreshing, heavy acid, (yogurt-like) sake aroma, alcohol, pickle-like, aged smell, mellow, full-bodied, heavy, strawberry, berry |
| Sweet/Honey | vanilla, caramel-like, sugar incense, sugar, sticky sweet, phenethyl-like, honey-like sweetness, sweet scent, heated candy, cotton candy |
| Foxy | total aroma of grapes (American grapes), grape odour |
| Muscat | total aroma of muscat |
| Fruity/Tropical | apple, aesthetic, green fruity, glue, tropical fruit, pineapple, strawberry, berry |
| Fig. S1 Flavour wheel created for table grapes and evaluation terms in each flavour descriptor | |

Fig. S2 Sensory evaluation sheet for table grapes used in this study.

| **Table S1 Total soluble solids (TSS, °Brix) and total acidity (TA, g/100 g tartaric acid conversion) of the 102 grape samples used in this work**. | | | | | | |
| --- | --- | --- | --- | --- | --- | --- |
| **Cultivars** | **2017** | | **2018** | | **2019** | |
|  | **TSS** | **TA** | **TSS** | **TSS** | **TSS** | **TA** |
| **Muscat of Alexandria** | 19.30 | 0.41 | 20.80 | 0.34 | 17.40 | 0.40 |
| **Muscat Hamburg** | 18.80 | 0.55 | 20.90 | 0.47 | 17.70 | 0.43 |
| **Neo Muscat** | 19.10 | 0.41 | 21.90 | 0.33 | 16.80 | 0.42 |
| **Shine Muscat** | 18.40 | 0.32 | 19.60 | 0.32 | 19.10 | 0.38 |
| **Hakunan** | 17.10 | 0.58 | 18.80 | 0.45 | 19.10 | 0.61 |
| **Muscat A petis Grains Rouge ①** | - | - | 18.00 | 0.46 | - | - |
| **Muscat A petis Grains Rouge ②** | - | - | 20.70 | 0.52 | 17.90 | 0.53 |
| **Muscat A petis Grains Blancs ①** | - | - | 18.00 | 0.47 | - | - |
| **Muscat A petis Grains Blancs ②** | - | - | 21.80 | 0.50 | 16.80 | 0.52 |
| **Benitaka** | - | - | 20.40 | 0.36 | - | - |
| **Katta Kurgan** | 15.70 | 0.40 | 16.70 | 0.36 | - | - |
| **Rizamat** | 16.90 | 0.28 | 20.70 | 0.25 | 18.50 | 0.39 |
| **Alphonse Lavallee** | 15.20 | 0.43 | 14.90 | 0.37 | 14.00 | 0.42 |
| **Rosaki** | 17.70 | 0.32 | - | - | - | - |
| **Parkent** | - | - | 13.50 | 0.37 | - | - |
| **Koshu** | - | - | 20.40 | 0.45 | 17.20 | 0.67 |
| **Buffalo ①** | 15.60 | 0.67 | 17.40 | 0.48 | 14.40 | 0.39 |
| **Buffalo ②** | - | - | 20.20 | 0.53 | 16.50 | 0.48 |
| **Sunny Rouge** | 18.90 | 0.55 | 18.30 | 0.42 | 20.40 | 0.48 |
| **Delaware** | 21.60 | 0.71 | 19.90 | 0.67 | 21.00 | 0.64 |
| **Muscat Bailey A** | 17.80 | 0.49 | 19.55 ^b^ | 0.45 ^b^ | 17.00 ^b^ | 0.49 ^b^ |
| **Oriental Star** | 18.80 | 0.46 | 20.40 | 0.35 | -^a^ | -^a^ |
| **Yuhou** | - | - | 13.50 | 0.22 | - | - |
| **Steuben ①** | 19.40 | 0.46 | 16.90 | 0.46 | 14.50 | 0.42 |
| **Steuben ②** | - | - | 21.60 | 0.48 | 17.50 | 0.55 |
| **Keuka** | 17.50 | 0.47 | 19.70 | 0.45 | 14.70 | 0.44 |
| **Honey Venus** | 20.50 | 0.50 | 21.70 | 0.47 | 19.70 | 0.47 |
| **Himrod** | 19.10 | 0.55 | 22.60 | 0.54 | 19.90 | 0.51 |
| **Sun Verde** | 19.80 | 0.43 | 20.20 | 0.36 | -^a^ | -^a^ |
| **Nagano Purple** | 21.60 | 0.47 | 21.20 | 0.37 | - | - |
| **Kyoho** | 19.70 | 0.49 | 18.40 | 0.47 | 20.30 | 0.57 |
| **Aki Queen** | 19.50 | 0.48 | 18.90 | 0.38 | 19.30 | 0.49 |
| **Queen Nina** | 21.60 | 0.36 | 20.80 | 0.39 | 20.80 | 0.50 |
| **Pione** | 20.70 | 0.44 | 21.50 | 0.41 | 20.40 | 0.46 |
| **Fujiminori** | 20.10 | 0.42 | 18.60 | 0.41 | -^a^ | -^a^ |
| **Black Beet** | 18.60 | 0.42 | 18.70 | 0.34 | 19.20 | 0.44 |
| **Campbell Early** | 15.20 | 0.61 | 16.90 | 0.56 | 13.60 | 0.62 |
| **Niagara** | 15.60 | 0.60 | 17.80 | 0.51 | 15.20 | 0.53 |
| ① and ② denote samples harvested from different trees in the same year.  ^-^ (without the superscript ^a^) Samples were not used for volatile component analysis and their TSS and TA were not measured  ^a^ Samples were used for volatile component analysis but their TSS and TA were not measured  ^b^ All values are quoted as the mean of the two samples harvested from two different trees. | | | | | | |

| **Table S2. Sensory evaluation scores between clusters based on Fig. 1.** | | | | | | | | | | | | | | |
| --- | --- | --- | --- | --- | --- | --- | --- | --- | --- | --- | --- | --- | --- | --- |
| **Flavour category** | **Cluster 1** | | **Cluster 2** | | **Cluster 3** | | **Cluster 4** | | **Cluster 5** | | **Cluster 6** | | **Cluster 7** | |
| **Intensity** | 3.42 ± 0.15 | ^BC^ | 1.29 ± 0.09 | ^D^ | 3.03 ± 0.13 | ^C^ | 3.4 ± 0.07 | ^BC^ | 3.88 ± 0.09 | ^B^ | 5.94 ± 0.17 | ^A^ | 6.05 ± 0.17 | ^A^ |
| **Muscat** | 4.93 ± 0.17 | ^A^ | 1.35 ± 0.10 | ^C^ | 1.75 ± 0.11 | ^BC^ | 1.90 ± 0.15 | ^B^ | 1.31 ± 0.03 | ^C^ | 1.47 ± 0.10 | ^BC^ | 1.40 ± 0.13 | ^BC^ |
| **Foxy** | 1.36 ± 0.08 | ^E^ | 1.21 ± 0.05 | ^E^ | 2.44 ± 0.13 | ^D^ | 3.85 ± 0.11 | ^C^ | 5.23 ± 0.08 | ^B^ | 6.25 ± 0.13 | ^A^ | 3.70 ± 0.05 | ^C^ |
| **Fresh green** | 3.15 ± 0.14 | ^A^ | 2.66 ± 0.15 | ^AB^ | 2.81 ± 0.17 | ^AB^ | 2.69 ± 0.20 | ^AB^ | 2.16 ± 0.15 | ^B^ | 2.19 ± 0.14 | ^B^ | 2.00 ± 0.11 | ^B^ |
| **Fatty green** | 1.79 ± 0.08 | ^D^ | 1.63 ± 0.08 | ^D^ | 2.54 ± 0.16 | ^C^ | 3.21 ± 0.18 | ^B^ | 3.05 ± 0.11 | ^BC^ | 4.09 ± 0.22 | ^A^ | 4.15 ± 0.24 | ^A^ |
| **Floral** | 3.67 ± 0.15 | ^AB^ | 1.93 ± 0.12 | ^C^ | 3.65 ± 0.20 | ^AB^ | 3.53 ± 0.13 | ^B^ | 3.56 ± 0.11 | ^B^ | 3.75 ± 0.22 | ^AB^ | 4.40 ± 0.22 | ^A^ |
| **Fermented / Sour** | 2.11 ± 0.09 | ^C^ | 1.63 ± 0.07 | ^C^ | 2.81 ± 0.18 | ^B^ | 3.10 ± 0.14 | ^B^ | 3.70 ± 0.11 | ^A^ | 4.16 ± 0.16 | ^A^ | 4.10 ± 0.10 | ^A^ |
| **Sweet** | 3.02 ± 0.14 | ^C^ | 2.07 ± 0.17 | ^D^ | 4.22 ± 0.17 | ^AB^ | 4.47 ± 0.12 | ^A^ | 4.17 ± 0.19 | ^AB^ | 3.56 ± 0.22 | ^BC^ | 5.00 ± 0.18 | ^A^ |
| **Fruity** | 2.90 ± 0.10 | ^D^ | 1.96 ± 0.13 | ^E^ | 3.57 ± 0.12 | ^C^ | 3.75 ± 0.13 | ^C^ | 4.03 ± 0.15 | ^BC^ | 4.69 ± 0.23 | ^B^ | 5.55 ± 0.20 | ^A^ |
| All values are quoted as the mean ± standard error. Any two samples with a common uppercase letter within each flavour category are not significantly different (*p-*value > 0.05) according to the Tukey-Kramer HSD test. | | | | | | | | | | | | | | |

| **Table S3** **Variable importance for projection (VIP) and coefficient (C) in each flavour category based on PLS analysis** | | | | | | | | | | | | | | | | | | |
| --- | --- | --- | --- | --- | --- | --- | --- | --- | --- | --- | --- | --- | --- | --- | --- | --- | --- | --- |
|  | **Intensity** | | **Muscat** | | **Foxy** | | **Fresh Green** | | **Fatty Green** | | **Floral** | | **Fermented / Sour** | | **Sweet** | | **Fruity** | |
|  | **VIP** | **C** | **VIP** | **C** | **VIP** | **C** | **VIP** | **C** | **VIP** | **C** | **VIP** | **C** | **VIP** | **C** | **VIP** | **C** | **VIP** | **C** |
| **hexanal** | 0.69 | -0.04 | 0.24 | 0.01 | 0.29 | -0.01 | 0.56 | 0.01 | 0.14 | 0.00 | 0.28 | -0.01 | 0.46 | -0.02 | 1.23 | -0.06 | 0.34 | -0.01 |
| **3-hexenal** | 1.33 | -0.06 | 0.67 | 0.00 | 1.10 | -0.03 | 0.95 | 0.01 | 0.89 | -0.02 | 0.68 | -0.01 | 1.38 | -0.05 | 0.81 | -0.03 | 1.11 | -0.02 |
| **(*Z*)-2-hexenal** | 0.48 | 0.02 | 0.47 | 0.01 | 0.71 | 0.02 | 0.37 | 0.00 | 1.04 | 0.02 | 0.76 | 0.02 | 0.57 | 0.02 | 0.90 | 0.05 | 0.61 | 0.01 |
| **(*E*)-2-hexenal** | 0.83 | -0.05 | 0.24 | 0.01 | 0.12 | 0.00 | 0.54 | 0.01 | 0.07 | 0.00 | 0.04 | 0.00 | 0.75 | -0.03 | 1.39 | -0.07 | 0.19 | 0.00 |
| **1-hexanol** | 1.06 | 0.03 | 0.45 | 0.02 | 0.50 | 0.01 | 0.94 | -0.01 | 0.82 | 0.01 | 1.11 | 0.02 | 0.60 | 0.02 | 0.43 | 0.01 | 0.88 | 0.02 |
| **3-hexenol** | 0.43 | 0.00 | 0.52 | 0.02 | 0.55 | 0.01 | 0.06 | 0.00 | 0.61 | 0.01 | 0.17 | 0.00 | 0.51 | -0.01 | 0.87 | -0.04 | 0.27 | 0.01 |
| **(*E*)-2-hexenol** | 1.12 | 0.04 | 0.45 | 0.00 | 0.97 | 0.03 | 0.92 | -0.01 | 1.14 | 0.02 | 1.41 | 0.03 | 0.84 | 0.02 | 0.78 | 0.03 | 1.29 | 0.02 |
| **isoamyl alcohol** | 0.55 | 0.00 | 0.19 | 0.00 | 0.28 | -0.01 | 0.37 | 0.00 | 0.47 | 0.01 | 0.45 | 0.01 | 0.31 | 0.00 | 0.31 | 0.01 | 0.50 | 0.01 |
| **2,3-butanediol (1)** | 0.68 | 0.03 | 0.22 | 0.00 | 0.71 | 0.03 | 0.46 | -0.01 | 0.77 | 0.01 | 0.29 | 0.01 | 0.63 | 0.02 | 0.36 | 0.02 | 0.43 | 0.01 |
| **octanol** | 1.39 | 0.03 | 0.43 | 0.00 | 0.45 | 0.00 | 1.04 | -0.01 | 1.08 | 0.02 | 1.40 | 0.03 | 0.82 | 0.02 | 0.93 | 0.04 | 1.51 | 0.03 |
| **2,3-butanediol (2)** | 0.25 | 0.01 | 0.39 | 0.01 | 0.24 | 0.01 | 0.10 | 0.00 | 0.02 | 0.00 | 0.23 | 0.00 | 0.26 | 0.01 | 1.01 | 0.05 | 0.21 | 0.00 |
| **benzyl alcohol** | 0.80 | -0.01 | 0.61 | -0.02 | 0.40 | 0.00 | 0.66 | -0.01 | 0.61 | 0.01 | 0.66 | 0.01 | 0.56 | 0.01 | 0.60 | 0.02 | 0.70 | 0.01 |
| **phenylethyl alcohol** | 1.45 | 0.03 | 0.82 | -0.01 | 1.40 | 0.03 | 0.97 | -0.01 | 1.74 | 0.03 | 1.50 | 0.03 | 1.51 | 0.05 | 1.13 | 0.03 | 1.66 | 0.03 |
| **1-dodecanol** | 0.50 | 0.03 | 0.29 | 0.00 | 0.71 | 0.03 | 0.43 | -0.01 | 0.48 | 0.01 | 0.21 | 0.00 | 0.93 | 0.04 | 0.31 | 0.01 | 0.37 | 0.01 |
| **phenol** | 0.72 | -0.01 | 0.50 | 0.00 | 0.68 | 0.01 | 0.28 | 0.00 | 0.74 | 0.01 | 0.96 | 0.02 | 0.66 | 0.00 | 0.65 | -0.01 | 0.89 | 0.02 |
| **methyl 3-hydroxybutanoate** | 1.59 | 0.07 | 0.86 | 0.00 | 1.24 | 0.03 | 0.61 | -0.01 | 1.72 | 0.03 | 1.36 | 0.03 | 1.39 | 0.04 | 1.12 | 0.04 | 1.61 | 0.03 |
| **methyl salicylate** | 0.75 | 0.01 | 0.78 | 0.00 | 1.10 | 0.02 | 0.29 | 0.00 | 1.27 | 0.02 | 0.60 | 0.01 | 1.10 | 0.03 | 0.77 | 0.00 | 1.03 | 0.02 |
| **methyl anthranilate** | 0.37 | 0.02 | 0.29 | 0.00 | 0.36 | 0.01 | 0.00 | 0.00 | 0.44 | 0.01 | 0.18 | 0.00 | 0.57 | 0.02 | 0.31 | 0.01 | 0.34 | 0.01 |
| **methyl *N*-formylanthranilate** | 1.10 | 0.05 | 0.57 | 0.00 | 1.17 | 0.04 | 0.61 | -0.01 | 1.05 | 0.02 | 0.22 | 0.00 | 0.96 | 0.03 | 1.31 | -0.05 | 0.77 | 0.01 |
| **ethyl butanoate** | 1.12 | 0.02 | 1.12 | -0.01 | 1.59 | 0.03 | 1.18 | -0.01 | 1.01 | 0.02 | 1.41 | 0.03 | 1.30 | 0.01 | 1.15 | 0.02 | 1.59 | 0.03 |
| **ethyl 2-methylbutanoate** | 0.53 | 0.01 | 0.84 | -0.01 | 1.26 | 0.03 | 0.87 | -0.01 | 0.53 | 0.01 | 0.60 | 0.01 | 0.87 | 0.02 | 0.84 | 0.03 | 0.78 | 0.01 |
| **ethyl pentanoate** | 0.93 | 0.03 | 0.90 | 0.00 | 1.56 | 0.04 | 0.94 | -0.01 | 0.83 | 0.01 | 0.87 | 0.02 | 1.09 | 0.02 | 1.02 | -0.02 | 1.07 | 0.02 |
| **ethyl 2-butenoate** | 1.38 | 0.03 | 1.18 | 0.00 | 1.73 | 0.04 | 1.38 | -0.02 | 1.33 | 0.02 | 1.53 | 0.03 | 1.50 | 0.03 | 1.27 | 0.03 | 1.71 | 0.03 |
| **ethyl hexanoate** | 1.37 | 0.04 | 1.17 | -0.01 | 1.79 | 0.04 | 1.49 | -0.02 | 1.23 | 0.02 | 1.72 | 0.04 | 1.48 | 0.02 | 1.26 | 0.03 | 1.81 | 0.03 |
| **ethyl heptanoate** | 1.35 | 0.05 | 0.87 | 0.00 | 1.64 | 0.05 | 1.38 | -0.02 | 1.19 | 0.02 | 0.88 | 0.02 | 1.29 | 0.03 | 1.28 | -0.04 | 1.23 | 0.02 |
| **ethyl octanoate** | 1.66 | 0.05 | 1.29 | 0.00 | 1.92 | 0.05 | 1.91 | -0.02 | 1.62 | 0.03 | 1.47 | 0.03 | 1.74 | 0.04 | 1.40 | 0.03 | 1.95 | 0.04 |
| **ethyl 3-hydroxybutanoate** | 1.70 | 0.05 | 1.21 | -0.01 | 1.84 | 0.04 | 1.75 | -0.02 | 1.65 | 0.03 | 1.92 | 0.04 | 1.76 | 0.04 | 1.44 | 0.04 | 2.00 | 0.04 |
| **ethyl nonanoate** | 0.94 | 0.03 | 0.74 | 0.00 | 1.26 | 0.03 | 1.08 | -0.01 | 1.11 | 0.02 | 0.61 | 0.01 | 1.14 | 0.03 | 0.98 | -0.02 | 1.00 | 0.02 |
| **ethyl decanoate** | 1.57 | 0.04 | 1.25 | -0.01 | 1.89 | 0.05 | 1.72 | -0.02 | 1.53 | 0.03 | 1.56 | 0.03 | 1.71 | 0.04 | 1.35 | 0.02 | 1.88 | 0.03 |
| **ethyl benzoate** | 0.95 | 0.03 | 0.83 | 0.00 | 1.37 | 0.04 | 0.51 | -0.01 | 1.17 | 0.02 | 0.67 | 0.01 | 1.26 | 0.04 | 0.97 | -0.02 | 1.11 | 0.02 |
| **ethyl trans-4-decenoate** | 1.40 | 0.05 | 1.01 | 0.00 | 1.60 | 0.04 | 1.75 | -0.02 | 1.35 | 0.02 | 1.05 | 0.02 | 1.54 | 0.04 | 1.23 | -0.01 | 1.30 | 0.02 |
| **ethyl 3-hydroxyhexanoate** | 1.53 | 0.06 | 1.16 | 0.00 | 1.93 | 0.05 | 2.04 | -0.03 | 1.46 | 0.03 | 1.39 | 0.03 | 1.61 | 0.04 | 1.19 | 0.02 | 1.67 | 0.03 |
| **ethyl trans-2-decenoate** | 0.57 | 0.02 | 0.73 | -0.01 | 1.07 | 0.02 | 1.36 | -0.02 | 0.62 | 0.01 | 0.51 | 0.01 | 0.90 | 0.02 | 0.77 | 0.02 | 0.75 | 0.01 |
| **ethyl benzeneacetate** | 0.84 | 0.02 | 0.98 | 0.00 | 1.61 | 0.04 | 0.68 | -0.01 | 1.04 | 0.02 | 0.49 | 0.01 | 1.14 | 0.02 | 0.96 | 0.00 | 0.96 | 0.02 |
| **ethyl salicylate** | 0.77 | 0.02 | 0.67 | 0.00 | 1.12 | 0.03 | 0.92 | -0.01 | 0.96 | 0.02 | 0.31 | 0.01 | 0.97 | 0.02 | 0.89 | -0.02 | 0.82 | 0.01 |
| **ethyl (*E,Z*)-2,4-decadienoate** | 0.99 | 0.03 | 1.08 | 0.00 | 1.74 | 0.05 | 0.98 | -0.01 | 1.15 | 0.02 | 1.19 | 0.02 | 1.35 | 0.03 | 1.06 | 0.02 | 1.25 | 0.02 |
| **ethyl cinnamate** | 1.56 | 0.04 | 0.66 | 0.01 | 0.92 | 0.02 | 0.90 | -0.01 | 1.40 | 0.02 | 0.89 | 0.02 | 1.13 | 0.03 | 1.23 | -0.04 | 1.33 | 0.02 |
| **butyl acetate** | 1.47 | 0.05 | 0.50 | 0.00 | 0.66 | 0.01 | 1.19 | -0.02 | 0.93 | 0.02 | 1.45 | 0.03 | 0.94 | 0.02 | 0.74 | 0.01 | 1.47 | 0.03 |
| **hexyl acetate** | 1.39 | 0.03 | 0.43 | 0.00 | 0.46 | 0.00 | 1.08 | -0.01 | 1.06 | 0.02 | 1.44 | 0.03 | 0.82 | 0.02 | 0.93 | 0.04 | 1.53 | 0.03 |
| **hexyl hexanoate** | 1.23 | 0.02 | 0.38 | 0.00 | 0.47 | -0.01 | 0.84 | -0.01 | 1.00 | 0.02 | 1.19 | 0.02 | 0.76 | 0.02 | 0.90 | 0.04 | 1.32 | 0.02 |
| **butyl octanoate** | 1.47 | 0.03 | 0.49 | 0.00 | 0.55 | 0.00 | 1.12 | -0.01 | 1.14 | 0.02 | 1.27 | 0.03 | 0.88 | 0.02 | 0.83 | 0.02 | 1.49 | 0.03 |
| **benzyl acetate** | 1.39 | 0.03 | 0.43 | 0.00 | 1.23 | 0.03 | 1.58 | -0.02 | 1.63 | 0.03 | 1.46 | 0.03 | 1.44 | 0.04 | 1.18 | -0.01 | 1.51 | 0.03 |
| **β-phenethyl acetate** | 1.91 | 0.06 | 0.82 | 0.00 | 0.45 | 0.00 | 1.05 | -0.01 | 1.08 | 0.02 | 1.39 | 0.03 | 0.82 | 0.02 | 0.93 | 0.04 | 1.78 | 0.03 |
| **hexyl octanoate** | 1.39 | 0.03 | 0.43 | 0.00 | 0.46 | 0.00 | 1.04 | -0.01 | 1.08 | 0.02 | 1.39 | 0.03 | 0.82 | 0.02 | 0.93 | 0.04 | 1.51 | 0.03 |
| **phenethyl hexanoate** | 1.39 | 0.03 | 0.43 | 0.00 | 0.46 | 0.00 | 1.04 | -0.01 | 1.09 | 0.02 | 1.38 | 0.03 | 0.82 | 0.02 | 0.93 | 0.04 | 1.51 | 0.03 |
| **phenylethyl octanoate** | 1.38 | 0.03 | 0.43 | 0.00 | 0.47 | 0.00 | 1.06 | -0.01 | 1.06 | 0.02 | 1.41 | 0.03 | 0.82 | 0.02 | 0.93 | 0.04 | 1.50 | 0.03 |
| **acetic acid** | 1.07 | 0.03 | 0.89 | -0.02 | 1.14 | 0.03 | 1.97 | -0.02 | 1.35 | 0.02 | 0.43 | 0.01 | 1.51 | 0.06 | 0.87 | 0.02 | 1.13 | 0.02 |
| **butanoic acid** | 0.41 | 0.00 | 0.70 | -0.01 | 0.93 | 0.02 | 1.27 | -0.02 | 0.34 | 0.01 | 0.48 | 0.01 | 0.60 | 0.00 | 1.09 | 0.05 | 0.69 | 0.01 |
| **hexanoic acid** | 0.69 | 0.02 | 0.41 | 0.02 | 0.34 | 0.00 | 0.19 | 0.00 | 0.62 | 0.01 | 1.36 | 0.03 | 0.47 | 0.01 | 0.86 | 0.04 | 0.79 | 0.01 |
| **octanoic acid** | 0.48 | 0.00 | 0.44 | -0.01 | 0.48 | 0.01 | 0.13 | 0.00 | 0.55 | 0.01 | 0.23 | 0.00 | 0.48 | 0.01 | 0.57 | 0.03 | 0.48 | 0.01 |
| **nonanoic acid** | 1.29 | -0.02 | 0.42 | 0.00 | 0.49 | 0.00 | 0.91 | 0.01 | 1.05 | -0.02 | 1.36 | -0.03 | 0.72 | -0.01 | 0.72 | -0.02 | 1.45 | -0.03 |
| **decanoic acid** | 0.74 | -0.02 | 0.73 | -0.03 | 0.31 | 0.01 | 0.17 | 0.00 | 0.01 | 0.00 | 0.46 | -0.01 | 0.19 | 0.01 | 0.73 | 0.04 | 0.08 | 0.00 |
| **benzoic acid** | 0.69 | 0.04 | 0.17 | 0.01 | 0.49 | 0.02 | 0.02 | 0.00 | 0.46 | 0.01 | 0.38 | 0.01 | 0.80 | 0.04 | 0.56 | 0.03 | 0.36 | 0.01 |
| **acetoin** | 1.00 | 0.02 | 0.67 | 0.00 | 1.20 | 0.03 | 0.53 | -0.01 | 1.39 | 0.02 | 1.28 | 0.03 | 1.21 | 0.04 | 0.86 | 0.02 | 1.32 | 0.02 |
| **benzaldehyde** | 0.42 | -0.01 | 0.24 | 0.01 | 0.27 | 0.00 | 0.12 | 0.00 | 0.33 | 0.01 | 0.21 | 0.00 | 0.67 | -0.03 | 0.97 | -0.05 | 0.45 | 0.01 |
| **phenylacetaldehyde** | 1.28 | 0.04 | 0.93 | -0.01 | 1.22 | 0.03 | 0.78 | -0.01 | 1.91 | 0.03 | 1.10 | 0.02 | 1.44 | 0.05 | 1.17 | 0.04 | 1.49 | 0.03 |
| **vanillin** | 1.10 | -0.04 | 0.51 | 0.00 | 0.69 | -0.01 | 1.01 | 0.01 | 0.99 | -0.02 | 1.71 | -0.04 | 0.90 | -0.03 | 1.37 | -0.07 | 1.47 | -0.03 |
| **methyl vanillate** | 0.52 | 0.03 | 0.27 | 0.01 | 0.62 | 0.03 | 0.35 | 0.00 | 0.23 | 0.00 | 1.00 | 0.02 | 0.67 | 0.03 | 1.40 | 0.07 | 0.44 | 0.01 |
| **mesifuran** | 1.58 | 0.07 | 0.76 | -0.01 | 1.18 | 0.04 | 1.22 | -0.02 | 1.53 | 0.03 | 0.80 | 0.02 | 1.30 | 0.04 | 0.85 | 0.02 | 1.23 | 0.02 |
| **γ-butyrolactone** | 0.81 | 0.02 | 0.74 | -0.01 | 1.12 | 0.03 | 0.50 | -0.01 | 1.25 | 0.02 | 1.33 | 0.03 | 1.15 | 0.04 | 1.37 | 0.07 | 1.08 | 0.02 |
| **γ-hexalactone** | 0.90 | 0.01 | 0.27 | 0.00 | 0.30 | -0.01 | 0.47 | -0.01 | 0.91 | 0.02 | 0.61 | 0.01 | 0.53 | 0.01 | 0.54 | 0.02 | 0.86 | 0.02 |
| **furaneol** | 1.48 | 0.06 | 0.75 | -0.01 | 0.99 | 0.03 | 0.67 | -0.01 | 1.91 | 0.03 | 0.94 | 0.02 | 1.46 | 0.05 | 1.07 | 0.05 | 1.30 | 0.02 |
| **γ-decanolactone** | 0.19 | 0.01 | 0.39 | -0.01 | 0.62 | 0.02 | 0.59 | -0.01 | 0.51 | 0.01 | 0.13 | 0.00 | 0.46 | 0.02 | 0.87 | 0.05 | 0.11 | 0.00 |
| **α-pinene** | 0.42 | 0.02 | 0.78 | -0.02 | 0.94 | 0.03 | 0.68 | -0.01 | 0.75 | 0.01 | 0.83 | 0.02 | 1.13 | 0.05 | 2.06 | 0.11 | 0.75 | 0.01 |
| **β-pinene** | 0.49 | 0.02 | 0.88 | -0.02 | 1.06 | 0.03 | 0.84 | -0.01 | 0.85 | 0.02 | 0.80 | 0.02 | 1.32 | 0.05 | 2.16 | 0.11 | 0.84 | 0.02 |
| **β-phellandrene** | 0.41 | -0.01 | 0.67 | 0.00 | 0.91 | 0.02 | 0.44 | -0.01 | 0.59 | 0.01 | 0.45 | 0.01 | 0.68 | 0.01 | 1.06 | 0.05 | 0.75 | 0.01 |
| **limonene** | 0.66 | 0.04 | 0.32 | 0.01 | 0.74 | 0.03 | 0.60 | -0.01 | 0.35 | 0.01 | 0.93 | 0.02 | 0.87 | 0.04 | 0.95 | 0.05 | 0.49 | 0.01 |
| **eucalyptol** | 0.63 | 0.03 | 0.36 | 0.01 | 0.51 | 0.01 | 0.79 | 0.01 | 0.76 | 0.01 | 1.16 | 0.02 | 0.86 | 0.03 | 0.48 | -0.01 | 0.59 | 0.01 |
| **(*Z*)-β-ocimene** | 0.46 | 0.01 | 0.91 | 0.02 | 0.59 | 0.00 | 0.42 | 0.01 | 0.42 | -0.01 | 0.03 | 0.00 | 0.54 | 0.00 | 0.61 | 0.02 | 0.35 | -0.01 |
| **γ-terpinene** | 0.35 | 0.01 | 0.53 | -0.01 | 0.73 | 0.02 | 0.16 | 0.00 | 0.95 | 0.02 | 0.75 | 0.02 | 0.99 | 0.04 | 0.95 | 0.05 | 0.69 | 0.01 |
| **(*E*)-β-ocimene** | 0.72 | 0.04 | 0.96 | 0.03 | 0.65 | 0.01 | 0.21 | 0.00 | 0.17 | 0.00 | 0.11 | 0.00 | 0.80 | 0.03 | 0.55 | 0.01 | 0.40 | -0.01 |
| ***cis*-rose oxide** | 0.89 | 0.03 | 1.95 | 0.06 | 1.08 | -0.01 | 1.16 | 0.01 | 1.02 | -0.02 | 1.35 | 0.03 | 1.05 | 0.01 | 1.00 | 0.01 | 0.41 | -0.01 |
| ***trans*-roseoxide** | 0.60 | 0.02 | 1.17 | 0.03 | 0.68 | -0.01 | 0.56 | 0.01 | 0.61 | -0.01 | 1.41 | 0.03 | 0.72 | 0.02 | 0.58 | 0.00 | 0.17 | 0.00 |
| ***trans*-linalooloxide (furanoid)** | 0.95 | 0.04 | 2.03 | 0.06 | 1.10 | -0.01 | 1.18 | 0.02 | 1.06 | -0.02 | 1.31 | 0.03 | 1.04 | 0.01 | 1.02 | 0.01 | 0.38 | -0.01 |
| ***cis*-linalooloxide (furanoid)** | 0.97 | 0.04 | 2.00 | 0.06 | 1.08 | -0.01 | 1.51 | 0.02 | 1.12 | -0.02 | 1.31 | 0.03 | 0.99 | 0.01 | 1.06 | 0.02 | 0.31 | -0.01 |
| **linalool** | 0.91 | 0.03 | 2.09 | 0.06 | 1.19 | -0.01 | 1.79 | 0.02 | 1.21 | -0.02 | 0.79 | 0.02 | 1.09 | -0.01 | 1.02 | -0.01 | 0.71 | -0.01 |
| **hotrienol** | 0.89 | 0.02 | 2.05 | 0.06 | 1.30 | -0.02 | 1.40 | 0.02 | 1.42 | -0.03 | 1.03 | 0.02 | 1.16 | -0.01 | 1.12 | 0.00 | 0.71 | -0.01 |
| **α-terpineol** | 0.84 | 0.04 | 1.40 | 0.05 | 0.56 | 0.01 | 1.07 | 0.01 | 0.59 | -0.01 | 1.43 | 0.03 | 0.80 | 0.03 | 1.17 | 0.05 | 0.10 | 0.00 |
| **α-citral** | 0.35 | -0.01 | 0.59 | 0.01 | 0.36 | 0.01 | 0.54 | 0.01 | 0.30 | -0.01 | 0.12 | 0.00 | 0.32 | 0.00 | 0.66 | 0.03 | 0.08 | 0.00 |
| ***trans*-linalooloxide (pyranoid)** | 0.94 | 0.03 | 2.22 | 0.07 | 1.36 | -0.02 | 1.81 | 0.02 | 1.44 | -0.03 | 1.01 | 0.02 | 1.22 | -0.01 | 1.13 | -0.01 | 0.78 | -0.01 |
| ***cis*-linalooloxide (pyranoid)** | 1.01 | 0.04 | 2.31 | 0.07 | 1.30 | -0.01 | 1.65 | 0.02 | 1.34 | -0.02 | 1.13 | 0.02 | 1.19 | 0.00 | 1.13 | 0.00 | 0.71 | -0.01 |
| **β-citronellol** | 0.35 | -0.01 | 0.59 | 0.01 | 0.36 | 0.01 | 0.56 | 0.01 | 0.29 | -0.01 | 0.13 | 0.00 | 0.32 | 0.00 | 0.66 | 0.03 | 0.08 | 0.00 |
| **nerol** | 0.71 | 0.02 | 1.60 | 0.05 | 0.95 | -0.01 | 1.02 | 0.01 | 0.92 | -0.02 | 1.16 | 0.02 | 0.94 | 0.02 | 0.79 | -0.01 | 0.35 | -0.01 |
| **guaniol** | 0.76 | 0.02 | 1.53 | 0.04 | 0.99 | -0.01 | 1.05 | 0.01 | 0.79 | -0.01 | 1.22 | 0.03 | 0.79 | 0.00 | 0.71 | -0.01 | 0.12 | 0.00 |
| **2,6-dimethyl-3,7-octadiene-2,6-diol** | 0.88 | 0.01 | 1.68 | 0.04 | 1.26 | -0.02 | 1.27 | 0.02 | 1.24 | -0.02 | 0.30 | 0.01 | 1.16 | 0.00 | 1.13 | 0.01 | 0.89 | -0.02 |
| **6,7-dihydro-7-hydroxylinalool** | 1.11 | 0.04 | 1.55 | 0.05 | 0.78 | -0.01 | 0.31 | 0.00 | 0.48 | -0.01 | 1.40 | 0.03 | 0.70 | 0.02 | 0.92 | 0.04 | 0.26 | 0.00 |
| **β-terpineol** | 0.73 | 0.04 | 1.39 | 0.05 | 0.60 | -0.01 | 1.64 | 0.02 | 0.51 | -0.01 | 0.76 | 0.02 | 0.56 | 0.00 | 0.78 | 0.02 | 0.28 | -0.01 |
| **2,6-dimethyl-1,7-octadiene-3,6-diol** | 0.97 | 0.04 | 2.13 | 0.07 | 1.09 | 0.00 | 1.41 | 0.02 | 1.06 | -0.02 | 0.95 | 0.02 | 1.01 | 0.00 | 1.18 | 0.03 | 0.55 | -0.01 |
| **8-hydroxylinalool** | 1.05 | 0.05 | 2.25 | 0.07 | 1.12 | 0.00 | 1.43 | 0.02 | 1.06 | -0.02 | 1.00 | 0.02 | 1.05 | 0.00 | 1.22 | 0.03 | 0.57 | -0.01 |
| **geranic acid** | 0.82 | 0.03 | 1.58 | 0.04 | 1.03 | -0.01 | 0.99 | 0.01 | 0.68 | -0.01 | 0.87 | 0.02 | 0.82 | 0.00 | 0.78 | 0.01 | 0.30 | -0.01 |
| **β-caryophyllene** | 0.59 | -0.02 | 0.70 | 0.01 | 0.74 | -0.02 | 0.60 | 0.01 | 0.96 | -0.02 | 0.16 | 0.00 | 0.61 | -0.01 | 1.10 | -0.05 | 0.65 | -0.01 |
| **α-caryophyllene** | 0.56 | 0.00 | 1.24 | 0.03 | 0.86 | -0.01 | 0.66 | 0.01 | 0.95 | -0.02 | 0.59 | 0.01 | 0.76 | 0.00 | 0.86 | -0.03 | 0.56 | -0.01 |
| **(*Z*)-β-farnesene** | 0.60 | 0.00 | 0.80 | 0.02 | 0.83 | -0.02 | 1.19 | 0.02 | 0.50 | -0.01 | 0.34 | -0.01 | 0.73 | 0.01 | 0.68 | 0.00 | 0.79 | -0.01 |
| **α-farnesene (1)** | 0.78 | 0.04 | 0.19 | -0.01 | 0.67 | 0.03 | 0.08 | 0.00 | 0.79 | 0.01 | 0.75 | 0.02 | 1.07 | 0.05 | 0.16 | 0.00 | 0.49 | 0.01 |
| **α-farnesene (2)** | 0.96 | 0.06 | 0.36 | 0.01 | 0.38 | 0.01 | 0.87 | 0.01 | 0.30 | 0.01 | 0.66 | 0.01 | 1.24 | 0.05 | 1.30 | 0.06 | 0.05 | 0.00 |
| **calamenene** | 0.74 | 0.04 | 0.45 | 0.01 | 0.51 | 0.01 | 0.04 | 0.00 | 0.05 | 0.00 | 0.38 | 0.01 | 0.80 | 0.03 | 0.37 | -0.02 | 0.05 | 0.00 |
| **β-damascenone** | 0.72 | -0.03 | 0.66 | -0.03 | 0.46 | -0.02 | 0.11 | 0.00 | 0.42 | -0.01 | 1.23 | -0.03 | 0.45 | -0.02 | 1.14 | -0.06 | 0.69 | -0.01 |
| **β-ionone** | 0.59 | -0.01 | 0.33 | 0.01 | 0.14 | -0.01 | 0.66 | 0.01 | 0.22 | 0.00 | 0.64 | 0.01 | 0.45 | -0.02 | 0.06 | 0.00 | 0.34 | 0.01 |

| **Table S4 Compound detection frequency (%) within each cluster based on Fig. 1.** | | | | | | | |
| --- | --- | --- | --- | --- | --- | --- | --- |
|  | **Cluster 1** | **Cluster 2** | **Cluster 3** | **Cluster 4** | **Cluster 5** | **Cluster 6** | **Cluster 7** |
| **hexanal** | 100 | 100 | 88.2 | 94.4 | 100 | 100 | 100 |
| **3-hexenal** | 100 | 100 | 76.5 | 100 | 75.0 | 50.0 | 60.0 |
| **(*Z*)-2-hexenal** | 9.52 | - | 17.6 | 33.3 | 18.8 | 50.0 | - |
| **(*E*)-2-hexenal** | 95.2 | 100 | 70.6 | 100 | 93.8 | 100 | 80.0 |
| **1-hexanol** | 90.5 | 58.8 | 52.9 | 66.7 | 93.8 | 87.5 | 100 |
| **3-hexenol** | 100 | 94.1 | 58.8 | 100 | 100 | 100 | 100 |
| **(*E*)-2-hexenol** | 52.4 | 47.1 | 58.8 | 83.3 | 93.8 | 100 | 100 |
| **isoamyl alcohol** | - | - | - | - | - | - | 20.0 |
| **2,3-butanediol (1)** | 9.52 | - | 23.5 | 5.56 | 6.25 | 62.5 | - |
| **octanol** | - | - | - | - | - | 12.5 | 100 |
| **2,3-butanediol (2)** | 28.6 | 5.88 | 23.5 | 5.56 | 25.0 | - | - |
| **benzyl alcohol** | 95.2 | 100 | 100 | 100 | 100 | 100 | 100 |
| **phenylethyl alcohol** | 71.4 | 64.7 | 70.6 | 100 | 100 | 100 | 100 |
| **1-dodecanol** | 23.8 | 23.5 | 47.1 | 83.3 | 62.5 | 62.5 | - |
| **phenol** | 57.1 | 58.8 | 58.8 | 66.7 | 100 | 87.5 | 100 |
| **methyl 3-hydroxybutanoate** | - | - | 23.5 | 50.0 | 25.0 | 100 | 100 |
| **methyl salicylate** | - | 5.88 | 17.6 | 38.9 | 50.0 | 75.0 | 20.0 |
| **methyl anthranilate** | - | - | 5.88 | 5.56 | 6.25 | 12.5 | - |
| **methyl *N*-formylanthranilate** | - | - | - | 5.56 | 6.25 | 75.0 | - |
| **ethyl butanoate** | - | 5.88 | 41.2 | 44.4 | 100 | 87.5 | 60.0 |
| **ethyl 2-methylbutanoate** | - | 5.88 | - | 27.8 | 100 | 12.5 | - |
| **ethyl pentanoate** | - | 5.88 | 5.88 | 22.2 | 81.3 | 87.5 | - |
| **ethyl 2-butenoate** | - | - | 29.4 | 44.4 | 100 | 87.5 | 80.0 |
| **ethyl hexanoate** | - | - | 41.2 | 44.4 | 100 | 100 | 60.0 |
| **ethyl heptanoate** | - | - | - | - | 56.3 | 100 | - |
| **ethyl octanoate** | - | - | 17.6 | 38.9 | 100 | 100 | 100 |
| **ethyl 3-hydroxybutanoate** | - | - | 29.4 | 55.6 | 100 | 100 | 100 |
| **ethyl nonanoate** | - | - | - | 11.1 | 37.5 | 62.5 | - |
| **ethyl decanoate** | - | - | 23.5 | 38.9 | 100 | 100 | 100 |
| **ethyl benzoate** | - | - | 5.88 | 22.2 | 56.3 | 75.0 | 20.0 |
| **ethyl trans-4-decenoate** | - | - | 5.88 | 11.1 | 75.0 | 87.5 | 40.0 |
| **ethyl 3-hydroxyhexanoate** | - | - | 5.88 | 38.9 | 93.8 | 100 | 40.0 |
| **ethyl trans-2-decenoate** | - | - | 5.88 | 16.7 | 62.5 | 12.5 | - |
| **ethyl benzeneacetate** | - | 5.88 | 11.8 | 33.3 | 87.5 | 75.0 | - |
| **ethyl salicylate** | - | 5.88 | 5.88 | 11.1 | 43.8 | 75.0 | - |
| **ethyl (*E,Z*)-2,4-decadienoate** | - | - | 29.4 | 61.1 | 100 | 87.5 | - |
| **ethyl cinnamate** | - | - | - | - | - | 75.0 | 80.0 |
| **butyl acetate** | - | - | - | - | - | 50.0 | 60.0 |
| **hexyl acetate** | - | - | - | - | - | 12.5 | 100 |
| **hexyl hexanoate** | - | - | - | - | - | - | 100 |
| **butyl octanoate** | - | - | - | - | - | 25.0 | 100 |
| **benzyl acetate** | - | - | - | - | - | 13 | 100 |
| **β-phenethyl acetate** | - | - | - | - | 37.5 | 100 | 100 |
| **hexyl octanoate** | - | - | - | - | - | 12.5 | 100 |
| **phenethyl hexanoate** | - | - | - | - | - | 12.5 | 100 |
| **phenylethyl octanoate** | - | - | - | - | - | 12.5 | 100 |
| **acetic acid** | 85.7 | 94.1 | 100 | 100 | 100 | 100 | 100 |
| **butanoic acid** | - | 5.88 | 11.8 | 27.8 | 68.8 | - | - |
| **hexanoic acid** | 95.2 | 82.4 | 82.4 | 94.4 | 93.8 | 100 | 100 |
| **octanoic acid** | 76.2 | 88.2 | 88.2 | 100 | 93.8 | 100 | 100 |
| **nonanoic acid** | 100 | 100 | 100 | 100 | 100 | 75.0 | - |
| **decanoic acid** | 42.9 | 58.8 | 88.24 | 100 | 100 | 37.5 | - |
| **benzoic acid** | 100 | 88.24 | 100 | 100 | 100 | 100 | 80.00 |
| **acetoin** | 76.2 | 70.6 | 70.6 | 94.4 | 100 | 100 | 100 |
| **benzaldehyde** | 90.5 | 94.1 | 70.6 | 94.4 | 100 | 100 | 100 |
| **phenylacetaldehyde** | - | - | 41.2 | 66.7 | 37.5 | 87.5 | 100 |
| **vanillin** | 100 | 100 | 100 | 94.4 | 100 | 87.5 | 60.0 |
| **methyl vanillate** | 38.1 | - | 70.6 | 66.7 | 31.3 | 50.0 | - |
| **mesifuran** | - | - | 35.3 | 38.9 | 6.25 | 100 | 100 |
| **γ-butyrolactone** | 19.0 | 11.8 | 52.9 | 83.3 | 75.0 | 75.0 | 60.0 |
| **γ-hexalactone** | - | - | - | - | - | - | 60.0 |
| **furaneol** | - | - | 41.2 | 61.1 | - | 100 | 100 |
| **γ-decanolactone** | - | - | - | 27.8 | 18.8 | - | - |
| **α-pinene** | 9.52 | - | 76.5 | 88.9 | 93.8 | 12.5 | - |
| **β-pinene** | 4.76 | - | 76.5 | 83.3 | 93.8 | 25.0 | - |
| **β-phellandrene** | - | - | 23.5 | 27.8 | 87.5 | - | - |
| **limonene** | 52.4 | 23.5 | 82.4 | 83.3 | 93.8 | 87.5 | - |
| **eucalyptol** | - | - | 5.88 | 11.1 | - | 37.5 | - |
| **(*Z*)-β-ocimene** | 42.9 | - | 17.6 | 27.8 | - | - | - |
| **γ-terpinene** | - | - | 47.1 | 55.6 | 31.3 | 50.0 | - |
| **(*E*)-β-ocimene** | 38.1 | - | - | 27.8 | - | - | - |
| ***cis*-rose oxide** | 76.2 | - | 11.8 | - | - | - | - |
| ***trans*-roseoxide** | 33.3 | - | 11.8 | - | - | - | - |
| ***trans*-linalooloxide (furanoid)** | 85.7 | - | 17.6 | 5.56 | - | 12.5 | - |
| ***cis*-linalooloxide (furanoid)** | 81.0 | - | 17.6 | 5.56 | - | 12.5 | - |
| **linalool** | 100 | 17.6 | 29.4 | 27.8 | - | 25.0 | - |
| **hotrienol** | 90.5 | 17.6 | 17.6 | - | - | - | - |
| **α-terpineol** | 100 | 17.6 | 47.1 | 83.3 | 81.3 | 37.5 | - |
| **α-citral** | 9.52 | - | - | - | - | - | - |
| ***trans*-linalooloxide (pyranoid)** | 100 | 17.6 | 17.6 | - | - | - | - |
| ***cis*-linalooloxide (pyranoid)** | 100 | 5.88 | 11.8 | - | - | - | - |
| **β-citronellol** | 9.52 | - | - | - | - | - | - |
| **nerol** | 61.9 | - | 17.6 | - | - | - | - |
| **guaniol** | 85.7 | 11.8 | 17.6 | - | - | 12.5 | 60.0 |
| **2,6-dimethyl-3,7-octadiene-2,6-diol** | 100 | 47.1 | 58.8 | 61.1 | 18.8 | 12.5 | - |
| **6,7-dihydro-7-hydroxylinalool** | 81.0 | 5.88 | 5.88 | 5.56 | - | 12.5 | 100 |
| **β-terpineol** | 23.8 | - | - | - | - | - | - |
| **2,6-dimethyl-1,7-octadiene-3,6-diol** | 76.2 | - | 5.88 | - | - | - | - |
| **8-hydroxylinalool** | 81.0 | - | - | - | - | - | - |
| **geranic acid** | 85.7 | 17.6 | 11.8 | 16.7 | - | - | 80.0 |
| **β-caryophyllene** | 38.1 | 23.5 | 17.6 | 5.56 | 12.5 | - | - |
| **α-caryophyllene** | 47.6 | 5.88 | 11.8 | - | - | - | - |
| **(*Z*)-β-farnesene** | 47.6 | 23.5 | 29.4 | 22.2 | - | - | - |
| **α-farnesene (1)** | 9.52 | 5.88 | 29.4 | 38.9 | - | 75.0 | - |
| **α-farnesene (2)** | 66.7 | 17.6 | 82.4 | 44.4 | 12.5 | 75.0 | - |
| **calamenene** | 100 | 82.4 | 76.5 | 88.9 | 81.3 | 100 | 100 |
| **β-damascenone** | 4.76 | 29.4 | 5.88 | 16.7 | - | - | - |
| **β-ionone** | 81.0 | 70.6 | 58.8 | 88.9 | 56.3 | 87.5 | 100 |
| ‘-‘ indicates that the compound was not detected in the sample. | | | | | | | |

| **Table S5 Average and standard error of OAV between clusters based on Fig. 1.** | | | | | | | | | |
| --- | --- | --- | --- | --- | --- | --- | --- | --- | --- |
| **OAV** | **Odour threshold (μg/l)** | | **Cluster 1** | **Cluster 2** | **Cluster 3** | **Cluster 4** | **Cluster 5** | **Cluster 6** | **Cluster 7** |
|  |  |  | **Mean**  **± SE** | **Mean**  **± SE** | **Mean**  **± SE** | **Mean**  **± SE** | **Mean**  **± SE** | **Mean**  **± SE** | **Mean**  **± SE** |
| **hexanal** | 4.5 | ^a^ | 5.67  ± 0.756 | 9.08  ± 2.57 | 5.46  ± 1.36 | 4.87  ± 0.898 | 3.59  ± 0.822 | 4.43  ± 0.926 | 3.64  ± 1.18 |
| **3-hexenal** | 0.25 | ^b^ | 722  ± 164 | 920  ± 188 | 596  ± 130 | 548  ± 105 | 177  ± 53.1 | 27.2  ± 9.69 | 22.0  ± 12.3 |
| **(*E*)-2-hexenal** | 17 | ^c^ | 8.10  ± 2.35 | 6.80  ± 1.47 | 2.85  ± 0.868 | 7.41  ± 1.48 | 4.98  ± 1.13 | 7.10  ± 2.15 | 1.07  ± 0.606 |
| **1-hexanol** | 500 | ^a,d^ | 0.0176  ± 0.00301 | 0.0121  ± 0.0037 | 0.0172  ± 0.00522 | 0.0179  ± 0.00454 | 0.0243  ± 0.00302 | 0.325  ± 0.00868 | 1.17  ± 0.302 |
| **3-hexenol** | 70 | ^a^ | 0.365  ± 0.118 | 0.245  ± 0.0565 | 0.553  ± 0.154 | 0.448  ± 0.111 | 0.691  ± 0.0781 | 0.65  ± 0.212 | 0.255  ± 0.0372 |
| **(*E*)-2-hexenol** | 100 | ^e^ | 0.0553  ± 0.0182 | 0.0838  ± 0.0393 | 0.125  ± 0.0738 | 0.989  ± 0.764 | 0.0556  ± 0.00909 | 0.464  ± 0.0321 | 0.559  ± 0.109 |
| **isoamyl alcohol** | 250 | ^f^ | - | - | - | - | - | - | 0.0127  ± 0.0127 |
| **2,3-butanediol (1)** | 2.6 | ^f^ | 0.634  ± 0.469 | - | 3.58  ± 1.94 | 2.31  ± 2.31 | 3.79  ± 3.79 | 125  ± 118 | - |
| **2,3-butanediol (2)** | 2.6 | ^f^ | 1.89  ± 0.760 | 0.0642  ± 0.0642 | 10.2  ± 7.11 | 6.16  ± 6.16 | 18.0  ± 11.6 | - | - |
| **benzyl alcohol** | 10000 | ^g^ | 0.00442  ± 0.00102 | 0.00581  ± 0.00143 | 0.00672  ± 0.0013 | 0.00429  ± 0.00059 | 0.00528  ± 0.00158 | 0.00986  ± 0.00143 | 0.0672  ± 0.0301 |
| **phenylethyl alcohol** | 1100 | ^g^ | 0.0135  ± 0.00363 | 0.0208  ± 0.0151 | 0.270  ± 0.196 | 0.296  ± 0.0571 | 1.23  ± 0.477 | 5.87  ± 1.72 | 31.2  ± 7.79 |
| **methyl anthranilate** | 3 | ^a^ | - | - | 0.0245  ± 0.0245 | 0.00788  ± 0.00788 | 0.00315  ± 0.00315 | 0.661  ± 0.706 | - |
| **ethyl butanoate** | 1 | ^a^ | - | 14.0  ± 14.0 | 27.8  ± 12.2 | 73.3  ± 29.1 | 163  ± 41.6 | 146  ± 30.5 | 21.2  ± 13.0 |
| **ethyl 2-methylbutanoate** | 0.1 | ^f^ | - | 11.8  ± 11.8 | - | 48.4  ± 26.1 | 194  ± 49.8 | 62.0  ± 66.3 | - |
| **ethyl hexanoate** | 1 | ^a^ | - | - | 8.35  ± 4.66 | 19.1  ± 8.01 | 80.1  ± 18.9 | 138  ± 33.9 | 23.3  ± 13.5 |
| **ethyl heptanoate** | 2 | ^a^ | - | - | - | - | 1.18  ± 0.344 | 3.27  ± 0.659 | - |
| **ethyl octanoate** | 194 | ^a^ | - | - | 0.0125  ± 0.0109 | 0.0517  ± 0.0266 | 0.135  ± 0.0304 | 0.488  ± 0.125 | 0.337  ± 0.161 |
| **ethyl 3-hydroxybutanoate** | 20000 | ^h^ | - | - | 0.000902  ± 0.000514 | 0.00466  ± 0.00137 | 0.0132  ± 0.00191 | 0.0377  ± 0.00459 | 0.0477  ± 0.0111 |
| **ethyl decanoate** | 88 | ^i^ | - | - | 0.0944  ± 0.0723 | 0.221  ± 0.126 | 0.565  ± 0.131 | 1.27  ± 0.418 | 0.201  ± 0.0543 |
| **ethyl 3-hydroxyhexanoate** | 63 | ^j^ | - | - | 0.00981  ± 0.00981 | 0.0649  ± 0.023 | 0.287  ± 0.0873 | 0.852  ± 0.434 | 0.316  ± 0.244 |
| **ethyl (*E,Z*)-2,4-decadienoate** | 2 | ^i^ | - | - | 41.4  ± 38.4 | 58.1  ± 35.3 | 68.9  ± 24.7 | 375  ± 332 | - |
| **ethyl cinnamate** | 1.1 | ^k^ | - | - | - | - | - | 13.8  ± 12.4 | 18.7  ± 6.49 |
| **butyl acetate** | 1500 | ^k^ | - | - | - | - | - | 0.0389  ± 0.000276 | 0.0588  ± 0.0334 |
| **hexyl acetate** | 1500 | ^k^ | - | - | - | - | - | - | 0.0303  ± 0.0155 |
| **β-phenethyl acetate** | 250 | ^h^ | - | - | - | - | 0.00688  ± 0.00504 | 3.69  ± 0.305 | 39.4  ± 9.30 |
| **acetic acid** | 144 | ^f^ | 0.135  ± 0.0420 | 0.0544  ± 0.0109 | 0.319  ± 0.176 | 0.345  ± 0.117 | 0.364  ± 0.14 | 1.46  ± 0.348 | 1.88  ± 0.663 |
| **butanoic acid** | 1000 | ^j^ | - | 0.000187  ± 0.000187 | 0.000146  ± 0.0001 | 0.00197  ± 0.000882 | 0.00548  ± 0.00262 | - | - |
| **hexanoic acid** | 3000 | ^o^ | 0.00525  ± 0.00109 | 0.00691  ± 0.00301 | 0.00873  ± 0.00331 | 0.00515  ± 0.000721 | 0.0111  ± 0.00464 | 0.00797  ± 0.00161 | 0.0150  ± 0.00583 |
| **octanoic acid** | 3000 | ^a^ | 0.0133  ± 0.0113 | 0.00282  ± 0.00126 | 0.00554  ± 0.00295 | 0.00274  ± 0.000695 | 0.00471  ± 0.00241 | 0.00244  ± 0.000607 | 0.00647  ± 0.00257 |
| **benzoic acid** | 1000 | ^k^ | 0.284  ± 0.244 | 0.0240  ± 0.00548 | 0.534  ± 0.325 | 0.0564  ± 0.0135 | 0.0466  ± 0.00902 | 0.0671  ± 0.00925 | 0.163  ± 0.0729 |
| **acetoin** | 8000 | ^a^ | 0.00375  ± 0.000888 | 0.00446  ± 0.00186 | 0.0183  ± 0.0101 | 0.0433  ± 0.00989 | 0.0797  ± 0.0113 | 0.0966  ± 0.0291 | 0.0284  ± 0.0116 |
| **benzaldehyde** | 350 | ^a^ | 0.0547  ± 0.0251 | 0.0418  ± 0.00855 | 0.0278  ± 0.00626 | 0.0367  ± 0.0119 | 0.0338  ± 0.0105 | 0.0566  ± 0.00637 | 0.0820  ± 0.0243 |
| **phenylacetaldehyde** | 4 | ^a^ | - | - | 3.12  ± 1.35 | 8.53  ± 2.76 | 2.62  ± 1.17 | 15.7  ± 3.33 | 70.0  ± 17.0 |
| **vanillin** | 60 | ^a^ | 1.95  ± 1.54 | 1.10  ± 0.355 | 1.78  ± 1.04 | 0.481  ± 0.117 | 0.196  ± 0.0423 | 0.298  ± 0.143 | 0.323  ± 0.185 |
| **methyl vanillate** | 3000 | ^k^ | 0.000428  ± 0.000145 | - | 0.00543  ± 0.00382 | 0.00177  ± 0.000826 | 0.00195  ± 0.00165 | 0.00424  ± 0.00353 | - |
| **mesifuran** | 16 | ^l^ | - | - | 11.1  ± 9.15 | 2.19  ± 1.17 | 0.191  ± 0.191 | 108  ± 63.3 | 3.30  ± 1.34 |
| **γ-butyrolactone** | 35 | ^k^ | 0.0771  ± 0.0544 | 0.0315  ± 0.0286 | 0.262  ± 0.100 | 0.503  ± 0.145 | 0.797  ± 0.388 | 0.327  ± 0.139 | 0.584  ± 0.441 |
| **furaneol** | 31 | ^f^ | - | - | 0.417  ± 0.313 | 10.2  ± 6.07 | - | 10.0 ± 4.76 | 6.74 ± 1.46 |
| **γ-decanolactone** | 88 | ^k^ | - | - | - | 0.0297  ± 0.0118 | 0.0121  ± 0.00893 | - | - |
| **α-pinene** | 6 | ^a^ | 0.0740  ± 0.0615 | - | 13.3  ± 6.70 | 17.0  ± 8.81 | 1.10  ± 0.517 | 0.200  ± 0.214 | - |
| **β-pinene** | 140 | ^a^ | 0.000270  ± 0.000270 | - | 0.563  ± 0.258 | 0.692  ± 0.325 | 0.0543  ± 0.0241 | 0.0138  ± 0.00951 | - |
| **limonene** | 10 | ^g^ | 2.32  ± 1.97 | 0.0519  ± 0.0293 | 2.46  ± 1.34 | 0.763  ± 0.31 | 0.266  ± 0.0885 | 0.110  ± 0.0476 | - |
| ***cis*-rose oxide** | 0.5 | ^g^ | 11.1  ± 2.69 | - | 2.75  ± 2.07 | - | - | - | - |
| ***trans*-roseoxide** | 0.5 | ^g^ | 1.71  ± 0.592 | - | 3.02  ± 2.56 | - | - | - | - |
| ***trans*-linalooloxide (furanoid)** | 320 | ^a^ | 0.0747  ± 0.0317 | - | 0.00395  ± 0.00247 | 0.000544  ± 0.000544 | - | 0.000405  ± 0.000433 | - |
| ***cis*-linalooloxide (furanoid)** | 320 | ^a^ | 0.0678  ± 0.0218 | - | 0.00447  ± 0.00319 | 0.000245  ± 0.000245 | - | 0.000333  ± 0.000356 | - |
| **linalool** | 6 | ^g^ | 76.9 ± 18.7 | 1.03  ± 0.828 | 0.576  ± 0.302 | 0.188  ± 0.0859 | - | 0.483  ± 0.336 | - |
| **hotrienol** | 110 | ^m^ | 0.814  ± 0.25 | 0.0229  ± 0.013 | 0.021  ± 0.0154 | - | - | - | - |
| **α-terpineol** | 330 | ^a^ | 0.555  ± 0.199 | 0.00942  ± 0.00553 | 0.0449  ± 0.0259 | 0.0264  ± 0.0136 | 0.0124  ± 0.00387 | 0.00407  ± 0.00217 | - |
| **α-citral** | 32 | ^a^ | 0.00955  ± 0.00778 | - | - | - | - | - | - |
| ***trans*-linalooloxide (pyranoid)** | 320 | ^a^ | 0.418  ± 0.121 | 0.00497  ± 0.00454 | 0.00405  ± 0.00278 | - | - | - | - |
| ***cis*-linalooloxide (pyranoid)** | 320 | ^a^ | 0.325  ± 0.154 | 0.000382  ± 0.000382 | 0.0023  ± 0.00158 | - | - | - | - |
| **β-citronellol** | 40 | ^g^ | 0.0642  ± 0.0579 | - | - | - | - | - | - |
| **nerol** | 300 | ^g^ | 0.0378  ± 0.0122 | - | 0.0133  ± 0.0107 | - | - | - | - |
| **guaniol** | 40 | ^g^ | 0.864  ± 0.253 | 0.0259  ± 0.0179 | 0.0489  ± 0.0338 | - | - | - | 0.166  ± 0.0914 |
| **geranic acid** | 40 | ^n^ | 3.67  ± 2.35 | 0.217  ± 0.122 | 0.190  ± 0.182 | 0.0129  ± 0.00778 | - | - | 0.776  ± 0.393 |
| **β-damascenone** | 0.002 | ^o^ | 70.2  ± 70.2 | 2350  ± 1320 | 320  ± 320 | 720  ± 460 | - | - | - |
| **β-ionone** | 0.007 | ^a^ | 829  ± 353 | 815  ± 210 | 379  ± 88.7 | 1410  ± 578 | 319  ± 196 | 879  ± 398 | 4890  ± 2460 |
| The concentration-odour threshold ratio, known as the ‘odour activity value’ (OAV), is commonly used to assess the contribution of each compound to fruit aroma. Compounds with OAVs greater than 1 are considered active contributors. Odour thresholds were obtained from the following literature. All compounds were quantified as 3-heptanol equivalents. "-" indicates that the compound was not detected in the sample.  a Pino, JA, Mesa, J. Contribution of volatile compounds to mango (*Mangifera indica* L.) aroma. *Flavour Fragr J* 2006; 21: 207–13, doi: 10.1002/ffj.1703  b Genovese, A, Dimaggio, R, Lisanti, MT, Piombino, P, Moio, L. Aroma composition of red wines by different extraction methods and Gas Chromatography-SIM/MASS spectrometry analysis. *Ann Chim* 2005; **95**: 383–94, doi: 10.1002/adic.200590045  c Genovese, A, Lamorte, SA, Gambuti, A, Moio, L. Aroma of Aglianico and Uva di Troia grapes by aromatic series. *Food* *Res* *Int* 2013; **53**: 15–23, doi: 10.1016/j.foodres.2013.03.051  d Buttery, R, Seifert, R, Guadagni, D, Ling, L. Characterization of additional volatile components of tomato. *J Agric Food Chem* 1971; **19**: 524–9, doi: 10.1021/jf60175a011  e Qian, MC, Wang, Y. Seasonal variation of volatile composition and odor activity value of ‘Marion’(Rubus spp. hyb) and ‘Thornless Evergreen’(*R. laciniatus* L.) blackberries. *J Food Sci* 2005; **70**: C13–C20, doi: 10.1111/j.1365-2621.2005.tb09013.x  f Baek, HH, Cadwallader, KR, Marroquin, E, Silva, JL. Identiﬁcation of predominant aroma compounds in muscadine grape juice. *J Food Sci* 1997; **62**: 249–52  g Fenoll, J, Manso, A, Hellin, P, Ruiz, L, Flores, P. Changes in the aromatic composition of the *Vitis* *vinifera* grape Muscat Hamburg during ripening. *Food* *Chem* 2009; **114**: 420–8, doi: 10.1016/j.foodchem.2008.09.060  h González Álvarez, M, González-Barreiro, C, Cancho-Grande, B, Simal-Gándara, J. Relationships between Godello white wine sensory properties and its aromatic fingerprinting obtained by GC-MS. *Food* *Chem* 2011; **129**: 890–8, doi: 10.1016/j.foodchem.2011.05.040  i Philipp, C, Eder, P, Brandes, W, Patzl-Fischerleitner, E, Eder, R. The pear aroma in the Austrian Pinot Blanc wine variety: Evaluation by means of sensorial-analytical-typograms with regard to vintage, wine styles, and origin of wines. *J Food Qual* 2018; 2018: 5123280, doi: 10.1155/2018/5123280  j Buettner, A, Schieberle, P. Evaluation of aroma differences between hand-squeezed juices from Valencia late and navel oranges by quantitation of key odorants and flavor reconstitution experiments. *J Agric Food Chem* 2001; **49**: 2387–94  k Cullere, L Escudero, A, Cacho, J, Ferreira, V.　Gas chromatography-olfactometry and chemical quantitative study of the aroma of six premium quality spanish aged red wines. *J Agric Food Chem* 2004; **52**: 1653–60  l Du, X, Plotto, A, Baldwin, E, Rouseff R. Evaluation of volatiles from two subtropical strawberry cultivars using GCOlfactometry, GC-MS odor activity values, and sensory analysis. *J Agric Food Chem* 2011; **59**: 12569–77  m Castro-Vázquez, L, Díaz-Maroto, M, Pérez-Coello, M. Aroma composition and new chemical markers of Spanish citrus honeys. *Food* *Chem* 2007; **103**: 601–6, doi: 10.1016/j.foodchem.2006.08.031  n Noguerol-Pato, R. *et al.* Floral, spicy and herbaceous active odorants in Gran Negro grapes from shoulders and tips into the cluster, and comparison with Brancellao and Mouratón varieties. *Food Chem* 2012; **135**: 2771–82, doi: 10.1016/j.foodchem.2012.06.104  o Buttery, RG, Teranishi, R, Ling, LC, Turnbaugh, JG. Quantitative and sensory studies on tomato paste volatiles. *J Agric Food Chem* 1990; **38**: 336–40, doi: 10.1021/jf00091a074 | | | | | | | | | |

**References**

1. Food and Agriculture Organization of the United Nations and the International Organisation of Vine and Wine. Table and Dried Grapes FAO FAO-OIV FOCUS 2016
2. Sun, Q, *et al*. Comparison of odor-active compounds in grapes and wines from *Vitis vinifera* and non-foxy American grape species. *J Agric Food Chem* 2011; **59**: 10657–64, doi: 10.1021/jf2026204
3. Klee, H, J, Improving the flavor of fresh fruits: genomics, biochemistry, and biotechnology. *New Phytol* 2010; **187**: 44–56, doi: 10.1111/j.1469-8137.2010.03281.x
4. Noble, A, C, *et al*. Modification of a standardized system of wine aroma terminology. *Am J Enol Vitic* 1987; **38.2**: 143–6, doi: 10.5344/ajev.1987.38.2.143
5. Vitis International Variety Catalogue. [www.vivc.de](file:///F:\投稿論文\www.vivc.de) (last accessed: July 15, 2023)
6. Yamada, M, Sato, A, Advances in table grape breeding in Japan. *Breed* *Sci* 2016; **66**: 34–45, doi: 10.1270/jsbbs.66.34
7. NARO Genebank. <https://www.gene.affrc.go.jp/about_en.php> (last accessed: July 15, 2023)
8. Yamada, M, *et al.* New grape cultivar ‘Honey Venus’. *Bull Natl Fruit Tree Res Stn* 2003; **2**: 53–63
9. Wu, Y, *et al.* Study on the volatile composition of table grapes of three aroma types. *LWT–Food Sci Technol* 2019; **115**: 108450, doi: 10.1016/j.lwt.2019.108450
10. Yamada, M, *et al.* New grape cultivar ‘Sunny Rouge’. *Bull. Natl. Fruit Tree Res Stn* 2003; **2**: 33–42
11. Sato, A, *et al.* New grape cultivar ‘Sun Verde’. *Bull Natl Fruit Tree Res Stn* 2014; **17**: 19–38
12. PVP Office at MAFF, JAPAN, Plant Variety Protection. http://www.hinshu2.maff.go.jp/en/en_top.html (last accessed: July 15, 2023)
13. Sato, A, *et al.* New grape cultivar ‘Queen Nina’. *Bull Natl Fruit Tree Res Stn* 2013; **15**: 21–37
14. Brown, K, *et al.* Consumer acceptability of fresh-market Muscadine grapes. *J* *Food Sci* 2016; **81**: S2808–16, doi: 10.1111/1750-3841.13522
15. Robinson, A, L, *et al.* Origins of grape and wine aroma. Part 1. Chemical compounds and viticultural impacts. *Am J Enol Vitic* 2014; **65**: 1–24, doi: 10.5344/ajev.2013.12070
16. Yang, C, *et al.* Volatiles of grape berries evaluated at the germplasm level by headspace-SPME with GC-MS. *Food* *Chem* 2009; **114**: 1106–14, doi: 10.1016/j.foodchem.2008.10.061
17. Ilc, T, Werck-Reichhart, D, Navrot, N, Meta-analysis of the core aroma compounds of grape and wine aroma. *Front* *Plant* *Sci* 2016; **7**: 1472, doi: 10.3389/fpls.2016.01472
18. Kalua, C, M, Boss, P, K, Evolution of volatile compounds during the development of cabernet sauvignon grapes (*Vitis vinifera* L.). *J Agric Food Chem* 2009; **57**: 3818–30, doi: 10.1021/jf803471n
19. Fenoll, J, *et al*. Changes in the aromatic composition of the *Vitis vinifera* grape Muscat Hamburg during ripening. *Food Chem* 2009; **114**: 420–8, doi: 10.1016/j.foodchem.2008.09.060
20. Wang, J, De Luca, V, The biosynthesis and regulation of biosynthesis of Concord grape fruit esters, including ‘foxy’ methylanthranilate. *Plant J* 2005; **44**: 606–19, doi: 10.1111/j.1365-313X.2005.02552.x
21. Baek, H, H, *et al.* Identification of predominant aroma compounds in muscadine grape juice. *J Food Sci* 1997; **62**: 249–52, doi: 10.1111/j.1365-2621.1997.tb03978.x
22. Sasaki, T, *et al*. Characterisation of ‘Ruby Roman’ table grapes (*Vitis labruscana* Bailey) by sensory evaluation and analysis of aroma and taste compounds. *Food Sci Technol Res* 2020; **26**: 423–33, doi: 10.3136/fstr.26.423
23. Wu, Y, *et al*. Aroma characterization based on aromatic series analysis in table grapes. *Sci Rep* 2016; **6**: 31116, doi: 10.1038/srep31116
24. Deng, H, *et al.* Comparison of the fruit volatile profiles of five Muscadine grape cultivars (*Vitis rotundifolia* Michx.) using HS-SPME-GC/MS combined with multivariate statistical analysis. *Front Plant Sci* 2021; **12**: 728891, doi: 10.3389/fpls.2021.728891
25. Engel, W, Bahr, W, Schieberle, P.,Solvent assisted flavour evaporation a new and versatile technique for the careful and direct isolation of aroma compounds from complex food matrices. *Eur Food Res Technol* 1999; **209**: 237–41, doi: 10.1007/s002170050486
26. Xu, Y, Fan, W, Qian, M, C, Characterization of aroma compounds in apple cider using solvent-assisted flavor evaporation and headspace solid-phase microextraction. *J Agric Food Chem* 2007; **55**: 3051–7, doi:10.1021/jf0631732
27. Lau, H, *et al.* Characterising volatiles in tea (*Camellia sinensis*). Part I: Comparison of headspace-solid phase microextraction and solvent assisted flavour evaporation. *LWT* 2018; **94**: 178–89, doi: 10.1016/j.lwt.2018.04.058
28. Tieman, D, *et al.* The chemical interactions underlying tomato flavor preferences. *Curr Biol* 2012; **22.11**: 1035–9, doi: 10.1016/j.cub.2012.04.016
29. Wang, J, Gambetta, J, M, Jeffery DW. Comprehensive study of volatile compounds in two Australian rosé wines: Aroma extract dilution analysis (AEDA) of extracts prepared using solvent-assisted flavor evaporation (SAFE) or headspace solid-phase extraction (HS-SPE). *J Agric Food Chem* 2016; **64.19**: 3838–48, doi: 10.1021/acs.jafc.6b01030
30. Triba, M, N, *et al.* PLS/OPLS models in metabolomics: the impact of permutation of dataset rows on the K-fold cross-validation quality parameters. *Molecular BioSystems* 2015; **11.1**: 13–9, doi:10.1039/c4mb00414k
31. Wold, S, Sjostrom, M, Eriksson, L, PLS-Regression: A basic tool of chemometrics. *Chemom Intell Lab Syst* 2001; **58**: 109–30, doi: 10.1016/S0169-7439(01)00155-1
32. Ribereau-Gayon, P, Boidron, J, N, Terrier A. Aroma of Muscat grape varieties. *J Agric Food Chem* 1975; **23**: 1042–7, doi: 10.1021/jf60202a050
33. Mateo, J, J, Jimenez, M, Monoterpenes in grape juice and wines. *J Chromatogr A* 2000; **881**: 557–67, doi:10.1016/s0021-9673(99)01342-4
34. Ruiz-Garcia, L, *et al.* Prediction of Muscat aroma in table grape by analysis of rose oxide. *Food Chem* 2014; **154**: 151–7, doi: 10.1016/j.foodchem.2014.01.005
35. Shure, K, B, Acree, T, E, in Fruit Flavor: Biogenesis, Characterization, and Authentication (eds. Rouseff, RL, Leahy, MM), 127–133 (American Chemical Society, 1995)
36. El Hadi, M, A, M, *et al.* Advances in fruit aroma volatile research. *Molecules* 2013; **18**: 8200–29, doi: 10.3390/molecules18078200
37. Lytra, G, *et* *al*. Distribution and organoleptic impact of ethyl 3-hydroxybutanoate enantiomers in wine. *J Agric Food Chem* 2015; **63**: 10484–91, doi: 10.1021/acs.jafc.5b04332
38. Sasaki, K, *et al.* Identification of furaneol glucopyranoside, the precursor of strawberry-like aroma, furaneol, in Muscat Bailey A. *Am J Enol Vitic* 2015; **66**: 91–4
39. Laing, D, G, *et al.* Quality and intensity of binary odor mixtures. *Physiol* *Behav* 1984; **33**: 309–19, doi: 10.1016/0031-9384(84)90118-5
40. Berglund, B, Berglund, U, Lindvall, T. Psychological processing of odor mixtures. *Psychol Rev* 1976; **83**: 432–41, doi: 10.1037/0033-295X.83.6.432
41. Lytra, G, *et al.* Impact of perceptive interactions on red wine fruity aroma. *J Agric Food Chem* 2012; **60**: 12260–9, doi: 10.1021/jf302918q
42. Ferreira, V, de-la-Fuente-Blanco, A, Sáenz-Navajas, M, P, A new classification of perceptual interactions between odorants to interpret complex aroma systems. Application to model wine aroma. *Foods* 2021; **10**: 1627, doi: 10.3390/foods10071627
43. Wallace, W, E, (director) Retention Indices, In: *NIST Chemistry WebBook*, NIST Standard Reference Database Number 69, Eds. Linstrom, P, J, Mallard, W, G, National Institute of Standards and Technology, Gaithersburg MD, USA, doi: 10.18434/T4D303 (retrieved July 26, 2021)
44. SAS Institute Inc. *The PLS Procedure*. In: SAS/STAT 14.3 User’s Guide, 2017, Cary, NC: SAS Institute
45. Tobias, R, D, An introduction to partial least squares regression. In: *Proceedings of the Twentieth Annual SAS Users Group International Conference*, 1995, pp. 1250–7
